# Late paleozoic climate revealed by coral fossil patterns

**DOI:** 10.1371/journal.pone.0290127

**Published:** 2023-08-15

**Authors:** Weijia Zhang, Jiaju Li, Xue Cheng, Hangjie Yu, Shaomin Cai

**Affiliations:** 1 Department of Mathematics, Physics and Information Sciences, Shaoxing University, Shaoxing, Zhejiang, China; 2 Department of AOP Physics, University of Oxford, Wellington Square, Oxford, United Kingdom; 3 Department of Physics, Sun Yat-sen University, Guangzhou, Canton, China; 4 Department of Medicine, Shaoxing University, Shaoxing, Zhejiang, China; Birbal Sahni Institute of Palaeosciences: Birbal Sahni Institute of Palaeobotany, INDIA

## Abstract

The study of coral fossils with clear growth lines inShiqiantan Formation (310 Ma) in China showed the growth line increments of a single coral growing in this area is related to the sunlight illumination time. Moreover, the daily increment of the growth line caused by calcium carbonate depositions has an unusual bimodal curve. The study of this bimodal curve indicated Shiqiantan had evident four-season changes in the Late Carboniferous period, and the climate at that time was similar to that of modern North China. The present paper updates the oldest existing record of climate analysis materials and provides important information for the study of Late Paleozoic climate, as well as the ancient obliquity of ecliptic.

## Introduction

Ancient sediments, shells, ice sheets, and rocks have long been regarded as valuable materials for reconstructing past climatic, environmental conditions and are important parts of paleoclimatology. The two main methods include measuring incremental growth patterns on fossil shells and analyzing oxygen isotope.

Oxygen isotope research is often used to reconstruct Cenozoic paleoclimates. For example, the isotope information on shells is the most reliable source of knowledge about seawater temperature conditions and the growth rates of shells [1].

The basic premise for measuring the growth increment pattern is that the fossil shell usually contains growth zones or lines with regular periodic precipitation. For example, Cudennec and Yves–Marie Paulet’s study confirmed the validity of the growth hypothesis by using calcein labeling experiment on Patella vulgata in the intertidal environment, and the growth of an individual reflects all the features encountered in the intertidal zone during growth [2].

Another promising finding of Wells [3] indicated the small ridges and annulations on the epitheca of corals might represent the daily and annual changes in skeletal deposition, respectively. According to Zhang et al. [4] and Moya et al. [5], such growth ridges are the results of light-enhanced calcification, and calcification rate is directly proportional to light intensity, thereby providing a theoretical basis for the study. This paper studies paleoclimate by considering light deposition calcification or growth line increments.

However, this method requires well-preserved, detailed subjects. The object of this paper is the coral fossil unearthed in the Shiqiantan Formation in Xinjiang Province. Only two intact samples were selected from the same batch of approximately 3100 coral fossils. The samples have very clear growth lines and can be used to study paleoclimate by measuring the growth increments.

Previous records of paleoclimatic reconstructions were held by ice sheets, and the oldest record is 5 million years old [6]. However, the fossils used in our paper remarkably exceed this record. The coral fossils in this paper were from the Shiqiantan Formation in Xinjiang Province obtained approximately 310 million years ago, a typical sedimentary layer from the Late Carboniferous, which preserved a diverse assemblage of coral fossils.

Research conducted by Wang [7] supports that coral fossils in the Shiqiantan Formation are predominantly found in shallow marine environments. This conclusion is based on the sensitivity of corals to environmental conditions and the geological context of the area [8]. However, subsequent to the deposition of the Shichantan Formation, corals and other shallow marine organisms gradually decreased and eventually became extinct in the Junggar region, suggesting a marine regression during the late stages of deposition, resulting in the transformation of the Junggar area from a shallow sea to a terrestrial environment.

## Materials and methods

**Fig 1:**
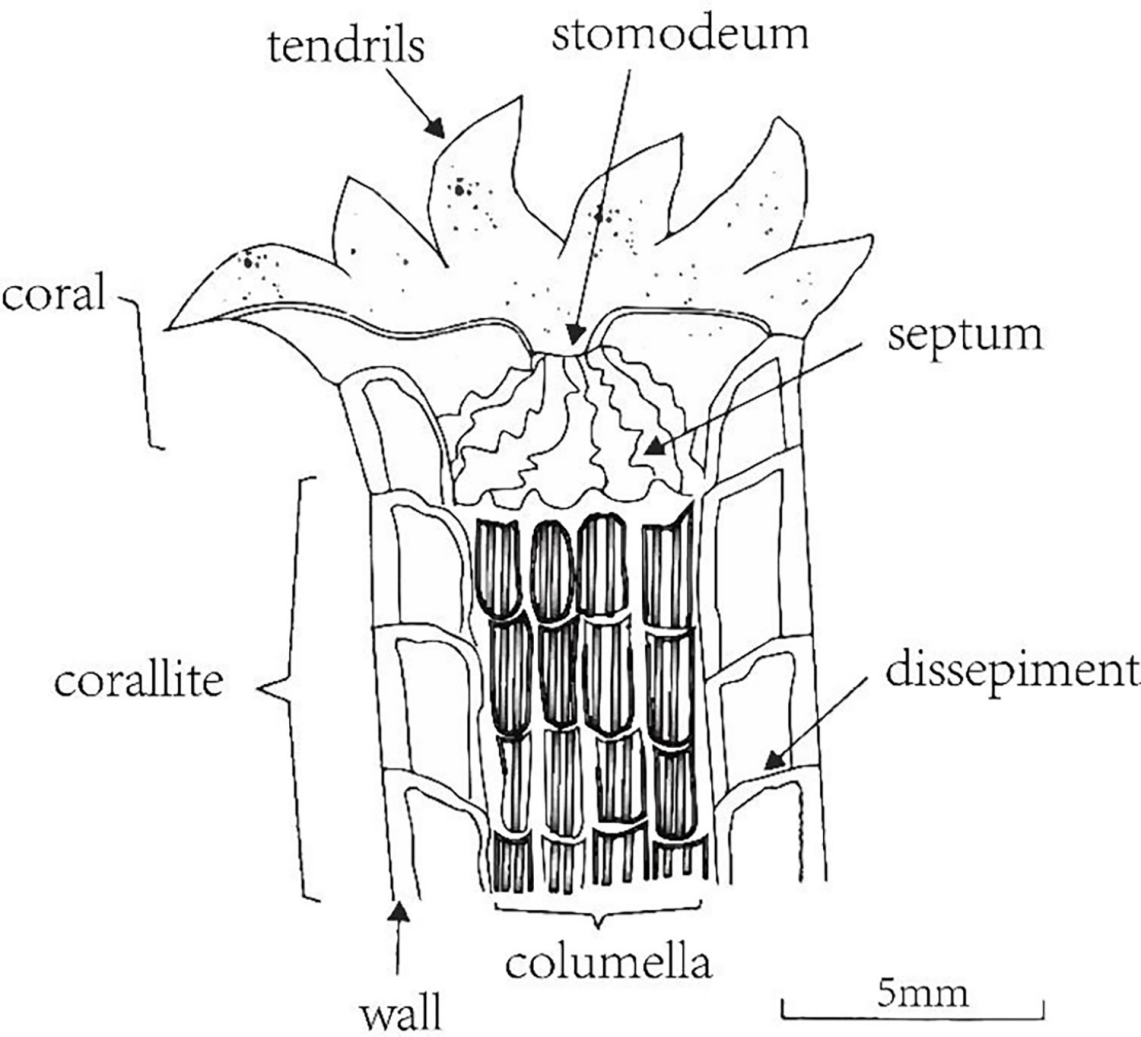
Basic structure of polyps and coral monomers [10].

This paper uses coral fossils unearthed in Shiqiantan in Xinjiang Province and belong to warm-water monomer corals [9]. The research on the specific structure and growth mechanism of the monomer coral is unavailable, but this ancient species of coral still exists. Thus, this paper starts with the relevant content of modern corals. The basic structure of modern coral skeleton includes calice, wall, septum, basal plate, dissepiment, columella, and spine, as shown in Fig 1.

**Fig 2:**
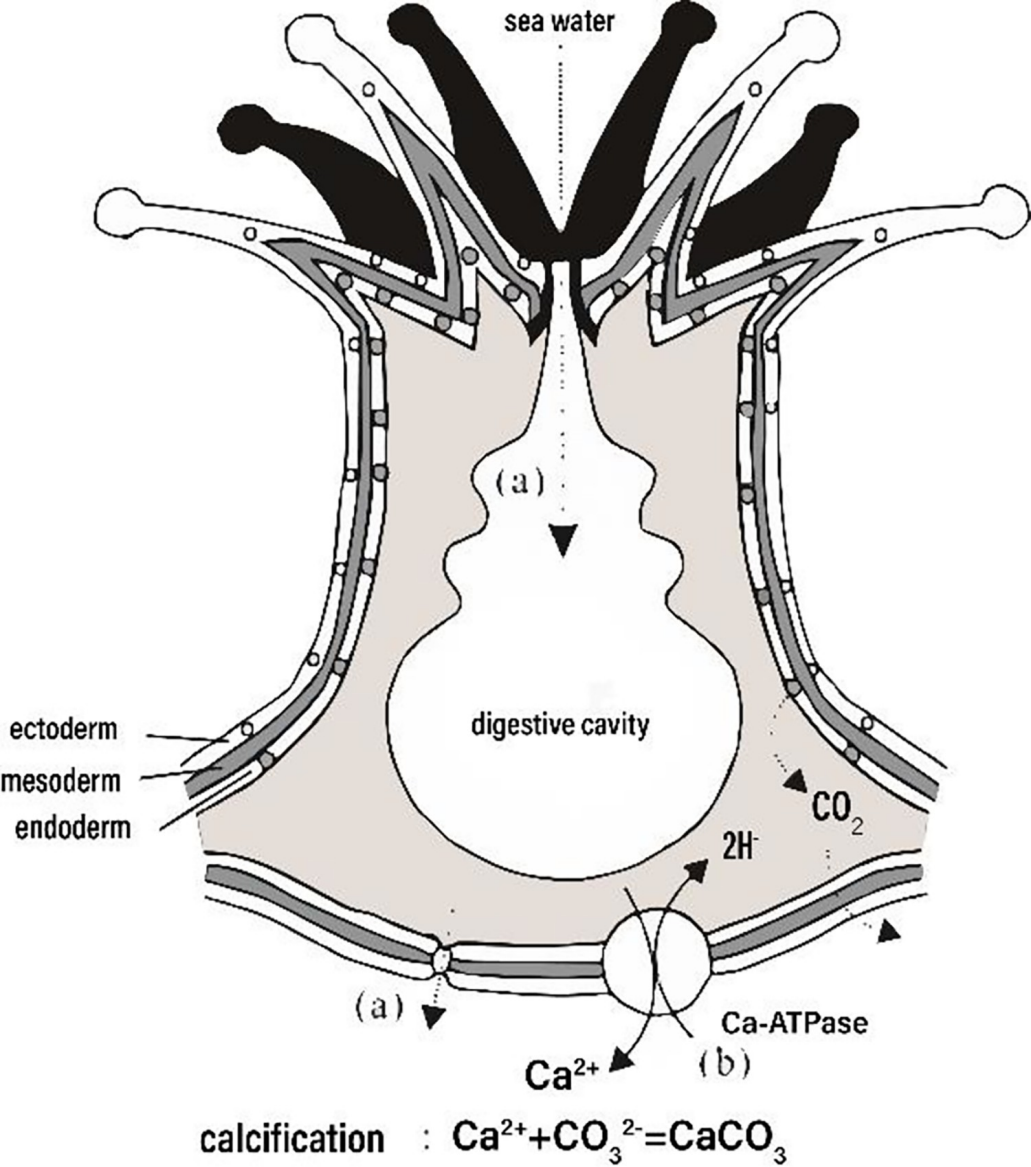
Elements enter the bone from seawater through calcified fluids and the main factors of element content [10].

Very fast-growing specimens are required to study climate variations through the measurement of growth line width; otherwise, the variation in width will not be distinguishable. Therefore, this paper starts with the growth pattern of hermatypic coral, as shown in Fig 2.

Hermatypic coral can efficiently use Ca2+ and dissolved inorganic carbon in seawater and rapidly precipitate aragonite calcium carbonate (CaCO3) skeletons, which are the main components of coral reefs.

Coral calcification is affected by light intensity. Eyal G. studied Euphylliaparadivisa [11] and found calcification increases dramatically with increasing light intensity in shallow-light-treated corals. This finding is consistent with the following calcification equation:

$${Ca}^{2+}+{2HCO}_{3}^{-}\overset{2Photon}{↔}{CaCO}_{3}+H_{2}{CO}_{3}$$ (1)

According to reaction kinetics, the reaction rate depends on the square of light intensity. The photons of the calcification equation are catalysts, and the study of Eyal G. Cohen I pointed out the minimum saturation irradiance. Thus, measurements of the width of the growth line can demonstrate the changes in day length or solar radiation. Although the fossil coral at Shiqiantan is not the type of hermatypic coral, the basic principle of light-enhanced calcification in coral species should not have changed, and the calcification deposits should follow the calcification equation. Therefore, the growth line width of coral fossils in the Shiqiantan Formation in Xinjiang Province can reflect the changes in ancient day length or solar radiation.

**Fig 3:**
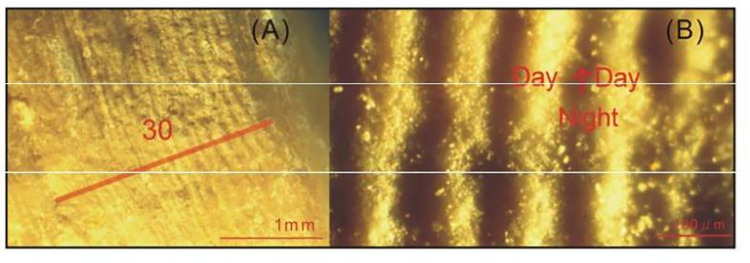
Diagram of growth ridges. A: A typical band on Carboniferous coral containing 30 ridges under a stereoscopic microscope. Specimen name: DZ-30-6; B: hundreds of ridges on our fossil coral epitheca observed under a stereoscopic microscope.

Growth ridges on fossil epitheca are a fundamental skeletal structure in tabulates rugosans and scleractinians. They are not only the basic materials of the exterior of the corallum but also the raw materials in various guises as septa and dissepiments, as shown in Fig 3.

Selecting the ideal sample and sampling path is the basic premise of coral research. The core drilled in this paper should be as close as possible to the direction of coral growth or at a small angle, so that the samples show clear bands of annual density.

**Fig 4:**
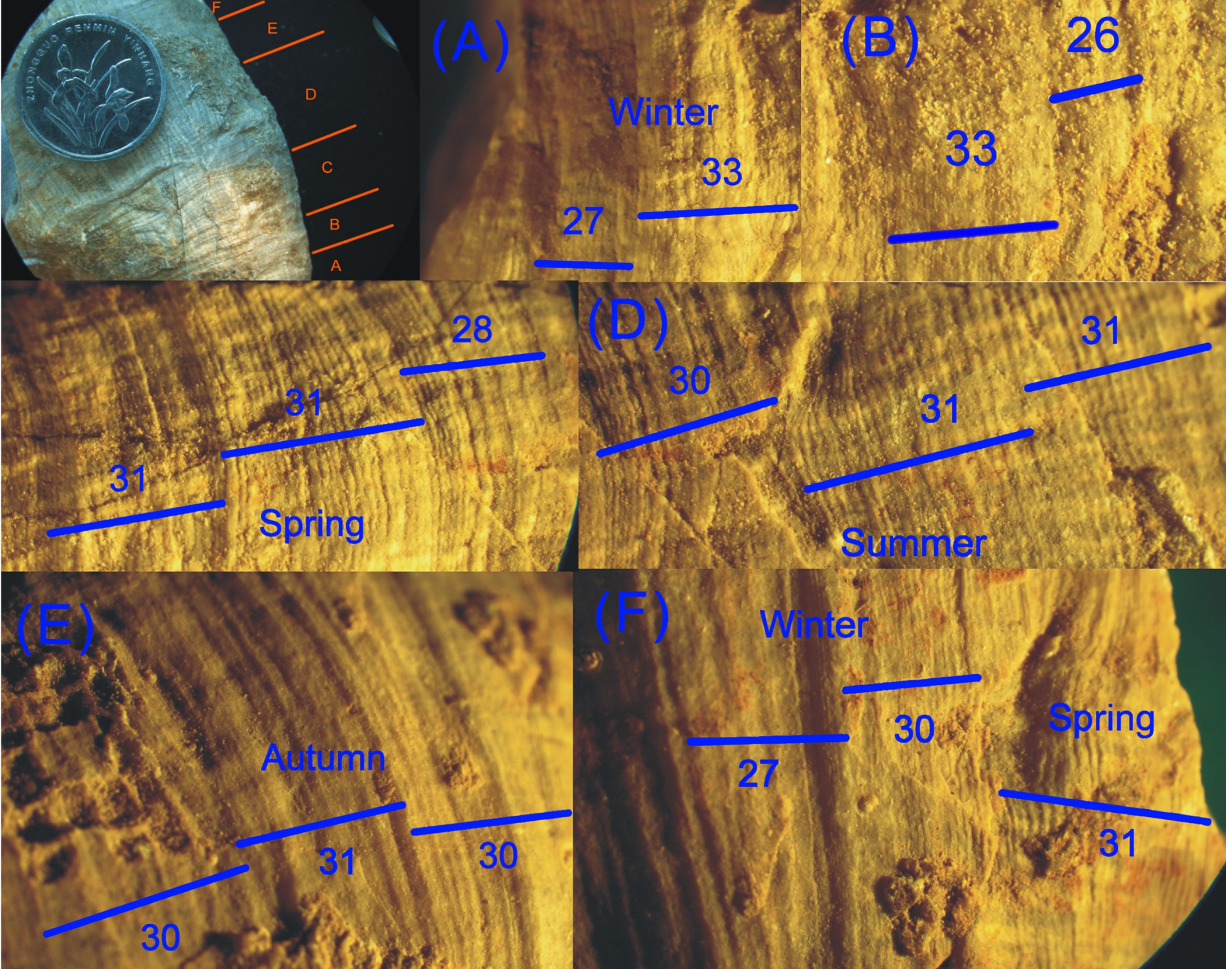
Diagram of coral fossil DZ-30-16 and enlargements of each part on coral epitheca (Late Carboniferous ~310 Ma, Shiqiantan Formation). More specimen details are included in the “S2 File”.

**Fig 5:**
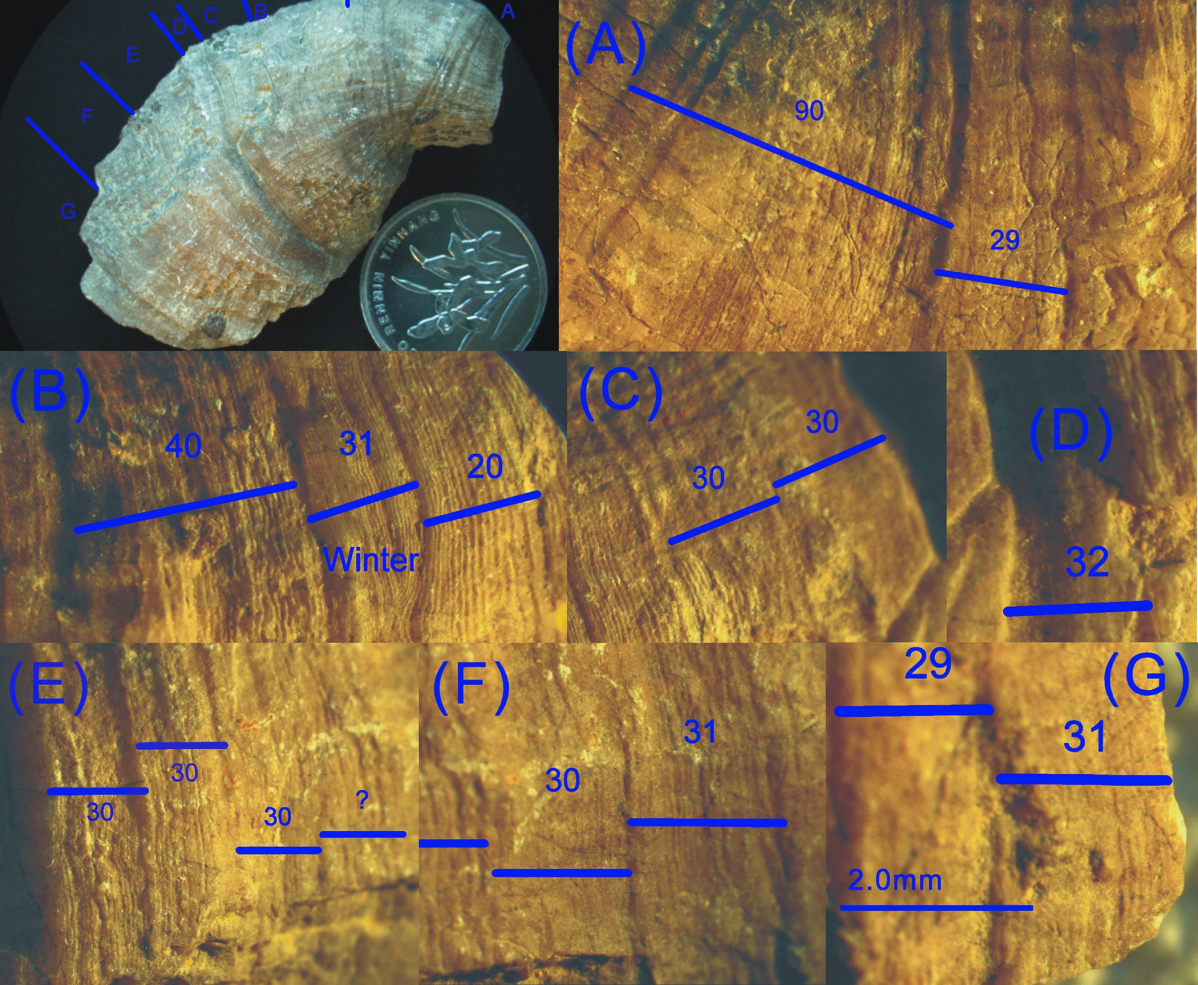
Diagram of coral fossil DZ-30-24 and enlargements of each part on coral epitheca (Late Carboniferous ~310 Ma, Shiqiantan Formation). More specimen details are included in the “S2 File”.

In this paper, two fast-growing samples with growth lines as wide as ~0.1 mm were selected. Every band on each specimen was presented in sequence from the left side of the coral fossil to the right side, with magnification under the PKU-SMZ1500 microscope. The number of ridges in each band was marked on the photographs and counted, and approximate maximum counts could be obtained. Two examples are shown in Figs 4 and 5. More measurement details are included in the “S1 File”.

## Results and discussion

### Bimodal phenomenon of the growth line width of the Late Carboniferous warm-water individual coral fossils

**Fig 6:**
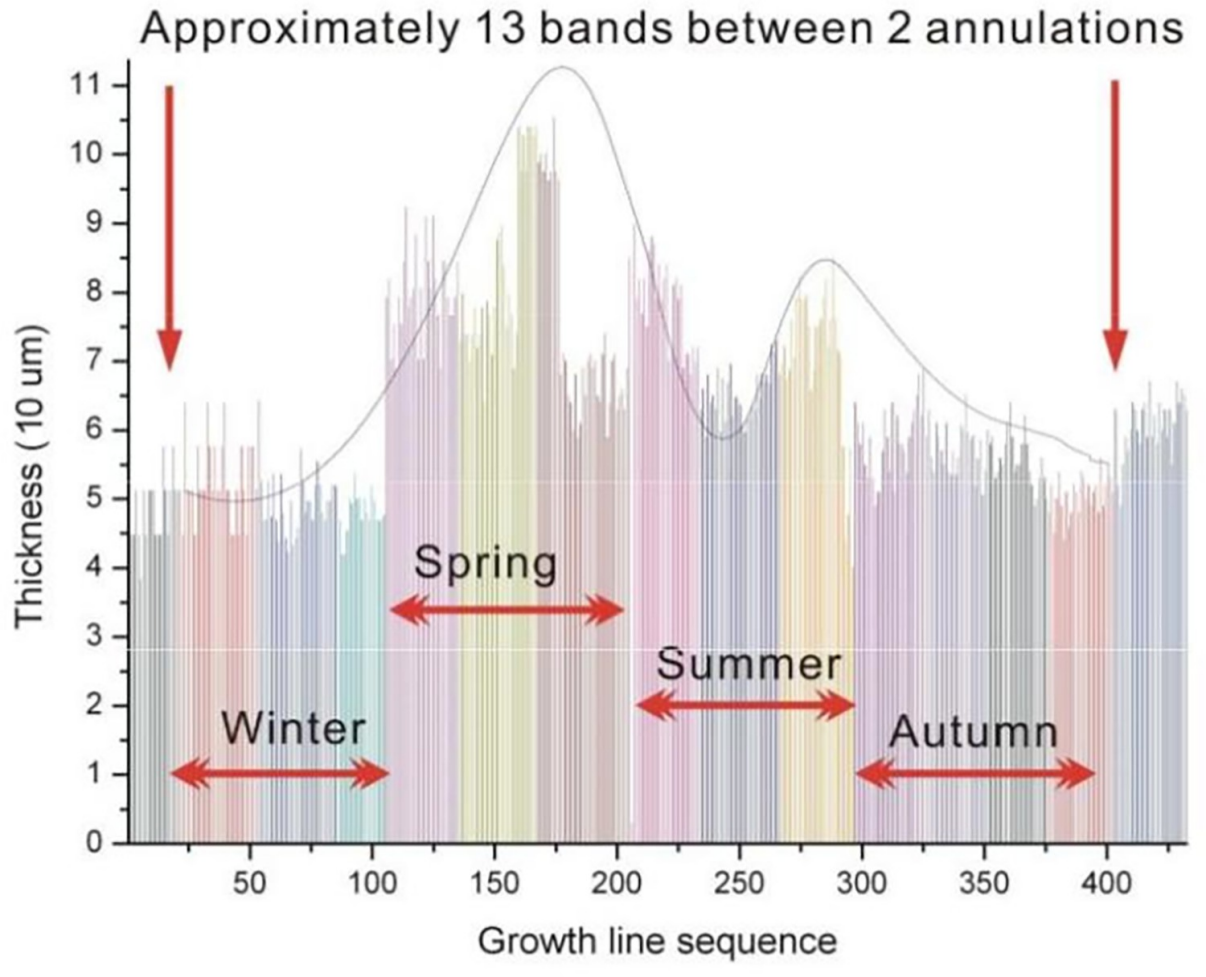
Width statistics of growth lines on fossil epitheca DZ-30-16, in sequence.

**Fig 7:**
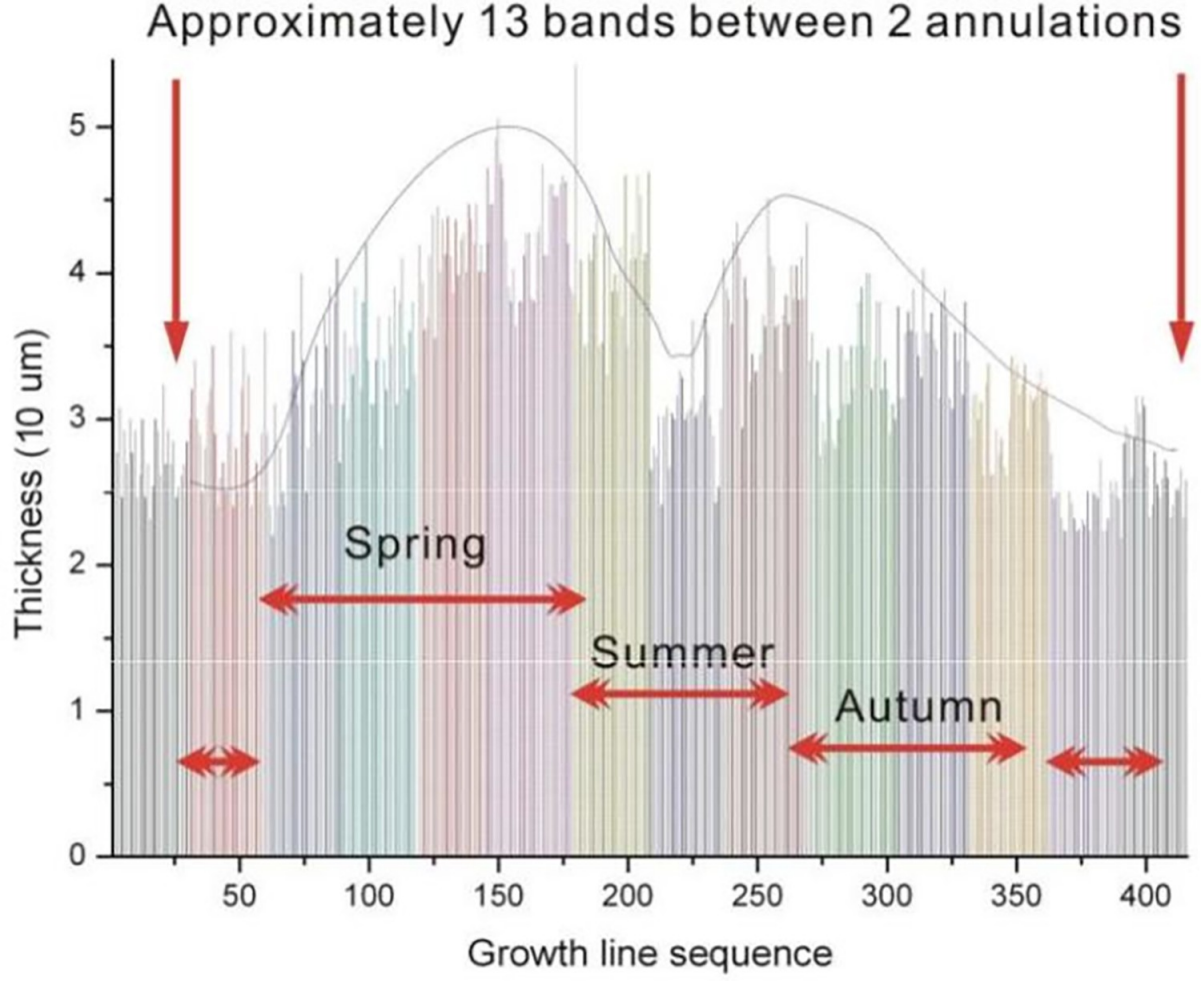
Width statistics of growth lines on fossil epitheca, in sequence.

The detailed statistical results on the width of the growth line are shown in Figs 6 and 7.

Fig 7 reveals four different intervals after dividing the graph into strips because the width of the growth lines in fossil corals can indicate changes in day length or solar radiation. Except in equatorial regions, the different illumination time in most areas of the four seasons inevitably affects the accumulation of calcification, resulting in different growth line widths in the four seasons. Therefore, the four sections of coral growth line width are caused by the evident differences in the four seasons in Shiqiantan in the Late Carboniferous period.

Unexpectedly, a peak was found in late spring, followed by a trough in midsummer, and another peak in late summer, after dividing the graph into four seasons. In addition, the first peak had a higher value than the second one.

The authors call it the bimodal phenomenon of the growth line width (of warm-water individual coral fossils in the Late Carboniferous), for which the reason remains unclear.

The Wilcoxon signed rank test, a commonly used nonparametric statistical test, was then used as a confidence interval analysis for such a bimodal phenomenon in the above figures. Its fundamental principle involves converting the difference between each paired sample into a signed rank and subsequently assessing whether a statistically significant difference exists between the two sample sets by comparing the sum of the signed ranks. One notable advantage of this method is its ability to accommodate small sample sizes and non-normally distributed data because it does not necessitate stringent assumptions about the data distribution [12].

Using a significance level of 0.05 and a sample size value of 20, with the Wilcoxon signed rank test, the values of the troughs of the DZ-30-16 and DZ-30-24 samples do not significantly exceed the values of the respective peaks. A high level of confidence (95%) supports the conclusion of a significant difference between the trough and peak data for the DZ-30-16 and DZ-30-24 samples.For the full analysisplease refer to the S3 File.

### Errors in the measurement

(1) Systematic error of the microscope

The total magnification rangeof the Nikon SMZ1500 microscope is 3.75–540X. According to the wave-particle duality of light, the resolution limit of the optical microscope can be obtained at 0.2 μm. While the measured growth line widths are all above 30 μm, it is far over the resolution limit of this optical microscope, so the growth line width can be observed using the SMZ1500.

To obtain accurate measurement data, magnification and external environmental conditions were kept consistent during the measurement until the measurement of all samples is completed.

(2) Measurement error

Another factor that affects the precision of the data is not intrinsically related to a faulty record but to our faulty interpretation of ambiguous patterns or to the difficulty of determining where to start and end counting patterns that usually merge into one another. This deviation is significantly reduced by 1) the ImagePro Plus (IPP) phase matching technique and modeling of coral patterns, as described in the “S1 File”, and 2) using only consecutive counts (at least three or four annual patterns).In this experiment, the SMZ1500 microscope was used to takehigh-precision pictures of fossil samples. Thepictures were magnified to observe the growth line of the coral fossil samples. A drawing tool and IPP phase matching software were used to determine the growth line width boundary of the sample.The average measurement error of 10 adjacent growth line widths on the sample was randomly calculated, and an estimated upper limit error value, which is about 5.5% of the growth line width, was obtained.

## Discussion

A theoretical derivation was conducted to explain the bimodal phenomenon.

**Fig 8:**
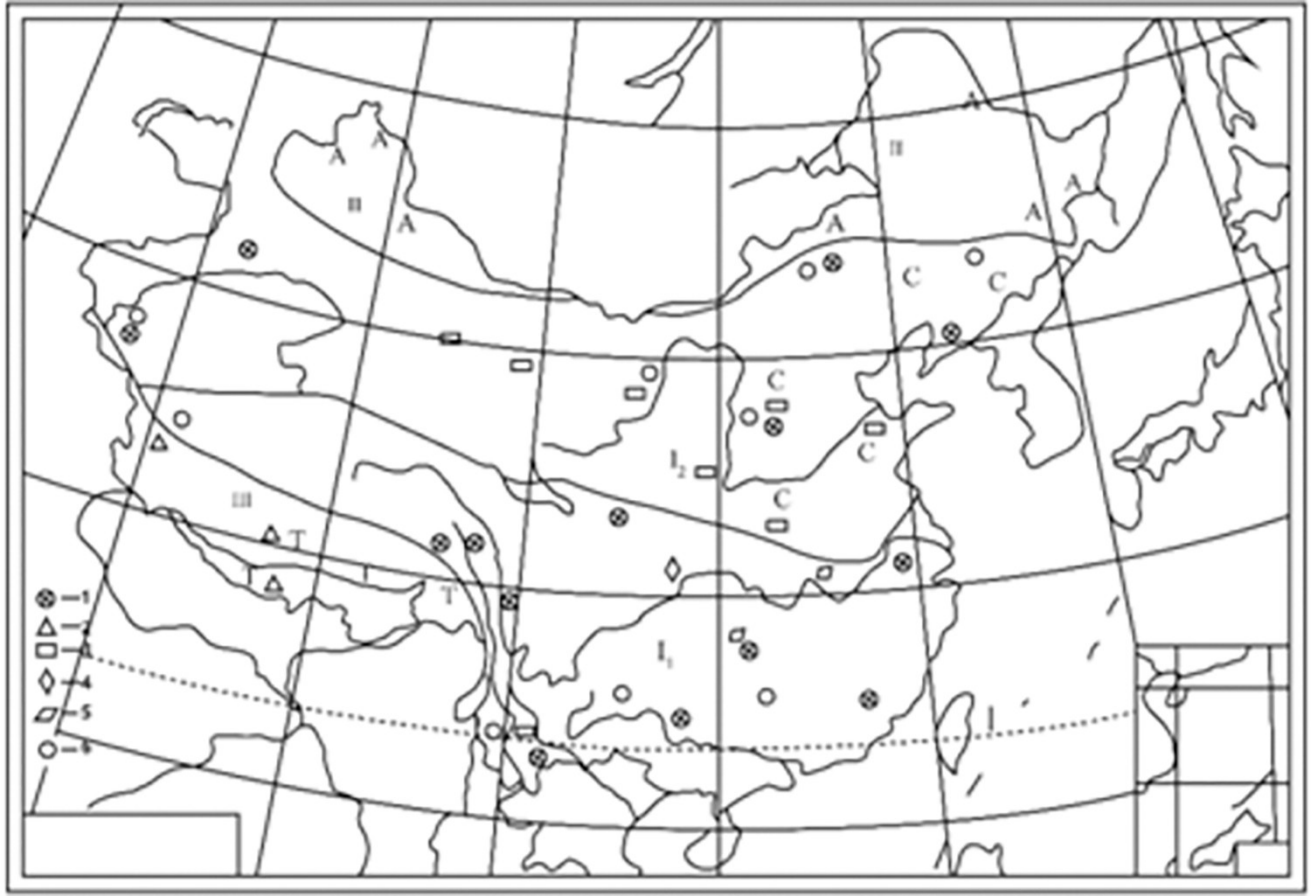
Late Carboniferous climatic zones of China. Reprinted from [13] under a CC BY license, with permission from [Earth Science], original copyright [1988]. Cathaysian tropical–subtropical region Ⅰ_1._ South China region Ⅰ_2._ North China subregion Ⅱ. Junggar-xing ’an North temperate zone Ⅲ. South temperate zone of southern Tibet A-Angara flora; C-Cathaysian flora; T-Glacial deposit 1-hermatypic coral; 2-cold-water fauna; 3-coal; 4-gypsum; 5-dolomite; 6-warm-water algae.

According to the research of Zhiping Wang and Fengqing Yang [13], Shiqiantan in Xinjiang Province belongs to the Junggar-Xing’an North temperate zone in the Carboniferous period, as shown in Fig 8.

**Fig 9:**
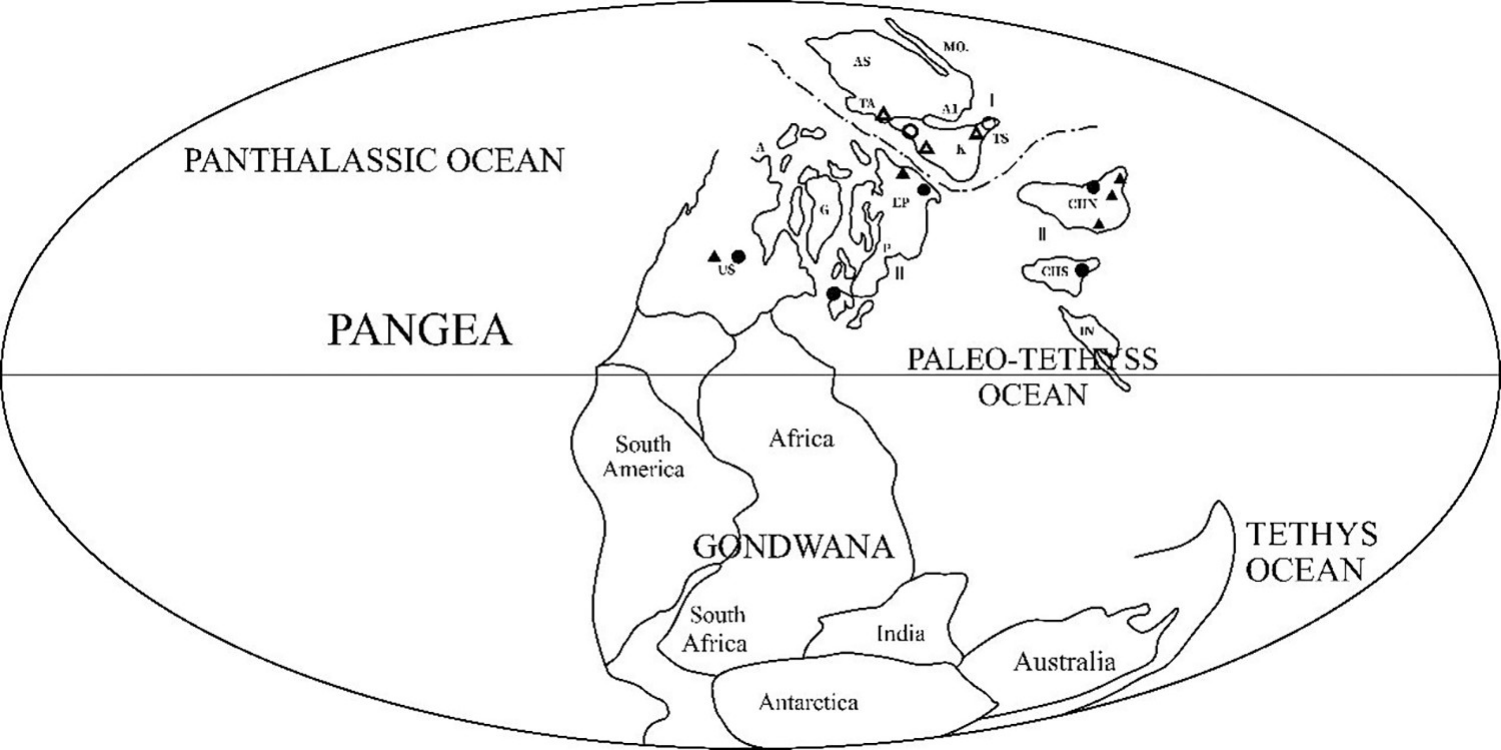
Paleobiogeography map of Middle and Carboniferous. Reprinted from [9] under a CC BY license, with permission from [Earth Science], original copyright [1992]. ⅠPaleobiog region; Ⅱ. Tethys region; A.Alaska,AI.Altai,As.North Asia; CHN. North China and Tarim; CHS.South China; Ep.Eastern Europe; G.Greenland; IN. Indochina; K.Kazakhstan; Mo.Mongolia; P.Poland; Ta.Temer; Ts.Tianshan Mountains; US.United States;▲ Low-latitude sea fan: Dumbarella, Pseudomontics; △ Mid-latitude sea fans: Altaipecten, Binipecten, “Annuliconcha;” ● Reef-building type coral: Petelaxis, Ivanovia; ◯ Warm-water monomer coral: Pseudotimania, Caninophyllum, Sestrophyllum.

Zhiping Wang and Qinglai Feng [14], based on the discussion of paleontological markers, stratigraphic characteristics, rock, ore markers, and oxygen isotope evidence, considered the position of the Junggar Plate at approximately 40°N–50°N. This finding indicates Shiqiantan was located at higher latitude in the Northern Hemisphere at that time. They presented the biological zoning maps of corals and bivalves in the Middle and Late Carboniferous period. The location of Shiqiantan in Xinjiang Province is shown by Ts in Fig 9.

The key findings of this short review are as follows: Shiqiantan belonged to a higher-latitude area in the Northern Hemisphere at that time. The sunshine duration at this location during the year changed with the seasons according to the current obliquity of the ecliptic. The increasing sunshine duration from January to April corresponded to longer sunshine duration during winter to spring in the Northern Hemisphere. At the end of summer, the days gradually shortened in the Northern Hemisphere due to seasonal changes, thereby gradually decreasing the number of monthly sunshine. The above conjecture can be verified by the sunshine duration in North China when the location is related to present-day North China.

**Fig 10:**
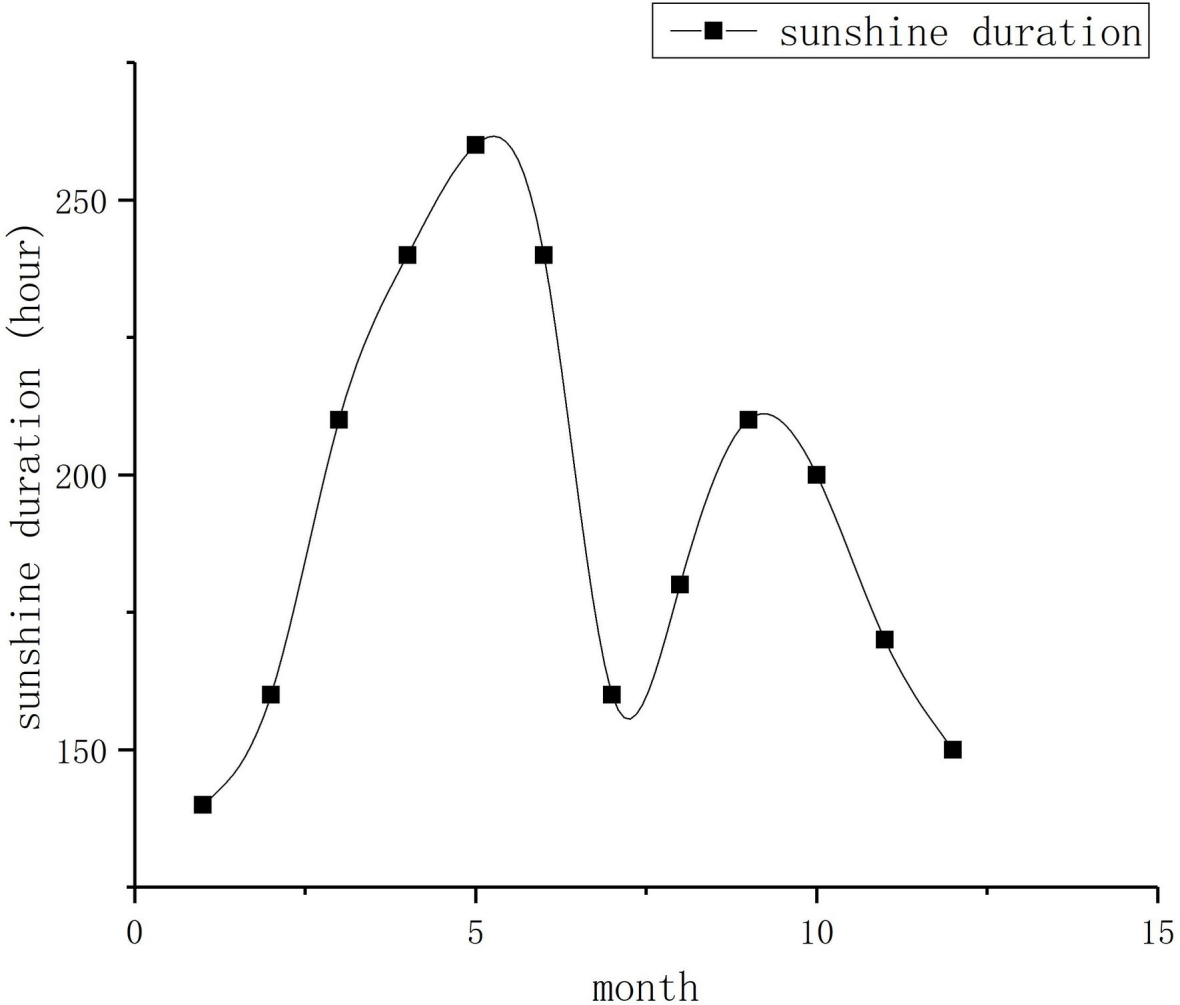
Schematic of the average monthly sunshine duration in Beijing (1981–2010).

Therefore, according to the monthly (annual) sunshine duration chart in the surface climate diagram of China (1981–2010) provided by the China Meteorological Data Service Center, the sunshine duration of January to December was obtained, as shown in Fig 10.

Chen [15] et al. established the mean sunshine duration time series of the North China Plain based on the sunshine duration data of 50 stations in the North China Plain provided by the China Meteorological Data Service Center. The monthly sunshine duration in descending order is May (252.8 h), June, April, August, July, March, September, October, November, January, February, and December (166.1 h). This result is consistent with that in Fig 10.

The results are very exciting. The variation of the monthly sunshine duration chart is relatively consistent with that of the coral growth line width, which clearly supports the positive correlation between the daily growth line width of individual corals in the Late Carboniferous and the sunshine duration. This finding suggests the growth line width of fossil corals can indicate the changes in day length. Therefore, the bimodal phenomenon of the growth line width of the warm-water individual coral fossils in the Late Carboniferous is positively correlated with the sunshine duration, and the climate of Shiqiantan in the Late Carboniferous is similar to that of North China, with an evident four-season variation, because these seasons and the climate in North China are derived from the obliquity of the ecliptic, indicating the obliquity of the ecliptic existed at that time, and the sample was located in a temperate zone. In addition, to form a temperate zone, the value of the ecliptic inclination must be less than 45°. According to Williams’ high-inclination hypothesis [16], the Ediacaran period was during 635–590 Ma, and the ecliptic inclination was about 54°. Considering the Late Carboniferous period of the sample was around 310 Ma, just between the present and the Ediacaran period, a simple median calculation can be performed, that is, the inclination of the ecliptic at that time may be around 38°, which is less than 45°.

In the S4 File, the possible theoretical variation of solar radiation intensity in the temperate zone in the Late Carboniferous is calculated based on the ecliptic angle of the Late Carboniferous at 38° assumed by Williams’research. The results are consistent with those obtained through specimen statistics.

## Conclusion

Our study of growth line width from fine coral fossils obtained important clues of Carboniferous period paleoclimate. Based on a detailed analysis of the growth increment of coral epithelium during the Carboniferous period, the following conclusions are drawn:

1. The hypothesis “the measurement of the width of the growth line can reflect the changes in solar day length” is again supported with new materials in this study.
2. The bimodal phenomenon of the growth line width of warm-water individual coral fossils in the Late Carboniferous period is positively correlated with the sunshine duration. Confidence interval analysis shows that the credibility of this result is above 95%.
3. The climate of Shiqiantan in the Late Carboniferous period was similar to that of modern North China, and there was a rainy season.
4. The seasonal changes in Shiqiantan area in the Late Carboniferous period were evident. Thus, the obliquity of the ecliptic, which causes Earth’s seasons, has already existed for at least 300 million years. In the supporting documents, we conducted a theoretical simulation of the sunshine variation calculated based on a 38° obliquity of the ecliptic, and the simulation results did not contradict the experimental observations.

After all, this is a preliminary study and should be regarded as an attempt. Further studies may either focus on dynamics or a search for new material. Although the dynamics of these materials are still being debated, they could provide an even longer time span for testing.

## Supporting information

S1 File Detailed methods and measurements. (PDF)

S2 File Specimen details. (PDF)

S3 File Confidence interval analysis. (PDF)

S4 File Effects of obliquity of the ecliptic and precipitation on sunshine duration during the Late Carboniferous. (PDF)
