# Supplementary material for: Late paleozoic climate revealed by coral fossil patterns
Source: PLoS One. 2023 Aug 15;18(8):e0290127. doi: 10.1371/journal.pone.0290127 (PMC10426913; doi:10.1371/journal.pone.0290127)
Supplement: S1 File — (PDF) [file pone.0290127.s001.pdf]

S1 File. Detailed Methods and Measurements

Data collection is restricted by the fact that the growth periodicity only exists on the epithecal surface. Therefore, research data have been derived from fossil growth increment records by counting features on specimens and in photographs. In this study, specimens were captured and examined with a scanning electron microscope, EMLPKU, or stereoscopic zoom microscope, SMZ1500. Distortion was reduced and chromatic aberration corrected to a high degree, resulting in natural-looking stereoscopic images. The counting involved either the direct recording of increments between successive higher order features or the continuous counting of increments over a number of cycles with the mean number of increments per cycle calculated by division, depending on the specimen's condition.

The 879 specimens selected from a total of 7000have clearly developed growth patterns on the epitheca and are among the most complex. Compared with former studies, we improved the manual counting and largely employed modern techniques, such as the use of an ImagePro Plus (IPP) processing facility and fast Fourier transform (FFT) techniques.

Principle

(1) Maximum count for each individual. It must be emphasized that the average count for each individual is subject to two factors. Firstly, under adverse conditions during the life of the organism involved only very limited secretion may occur..Secondly, the resulting growth lines may be obscure. Hence, there is inherent disagreement between the observed number of daily growth increments contained

within an annual zone on a particular fossil and the actual number of days per year in the period during which the organism lived. If used, the average count method would yield a minimum value for the number of days per month or year, while the maximum count method would overcome this systematic error and yield a higher, more accurate value.

(2) Mean-value calculation for all individuals. The maximum count for an individual could overcome the error caused by fossil abrasion while the mean-value calculation for all individuals could overcome the error in counting.

(3) Consecutive counting. A factor that affects the precision of the data is the difficulty of determining where to start and finish counting patterns that usually merge into each other. This difficulty is partly eliminated using only consecutive counts (at least three or four). It must be emphasized that the longer the sequence of consecutive counts, the more reliable the data (derived by dividing the total number of lines of the sequence by the number of periodical patterns). In this way, the subjective decision of the limits of the patterns is reduced to the two extreme patterns of the series. Fossils with more than four consecutive annulations were chosen and analyzed.

General procedures and pattern information recovery technologies

We attempted to improve measurements in the following waves.

(1) Each lunar band was analyzed under the scanning electron microscope EMLPKU or stereoscopic zoom microscope SMZ1500 and pictures were taken with sufficient amplification to exactly distinguish each diurnal ridge.

(2) IPP processing facility from Germany was used to eliminate abrasion and recover the specimen information, and the FFT could eliminate abrasion frequencies and recover ridge patterns. First, a photograph of patterns was converted into a monochrome figure. Second, the FFT extracted a detailed frequency distribution from this monochrome figure. Because the frequency of growth lines was centralized, at the same magnitude, the noise frequency which frets growth lines can be determined by trial and error. The researcher simply deleted the suspected area of noise using the IPP, and then applies an inverse FFT to see whether the growth lines were recovered without abrasion. Finally, the noise that distorted the pattern most significantly was removed, leaving the growth pattern clear again, like it was millions of years ago (Fig S1).

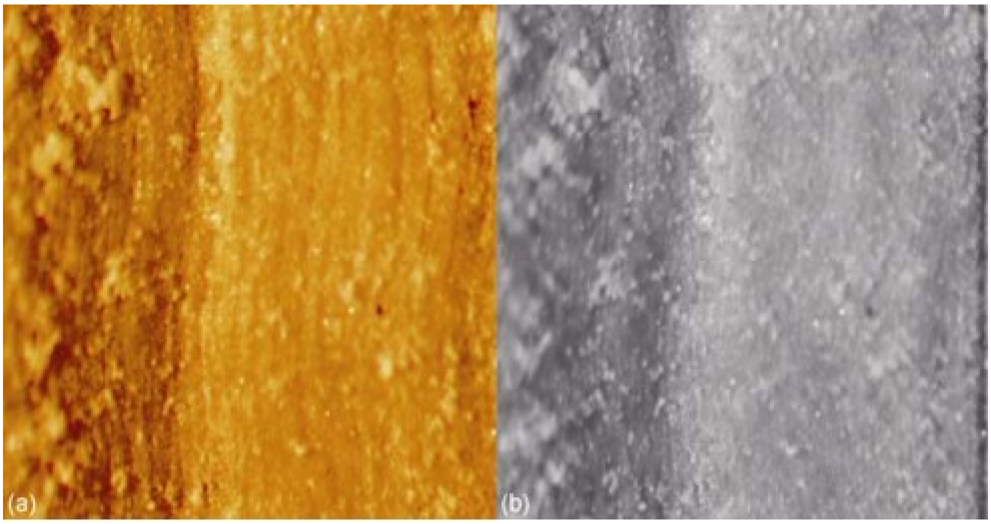

FigS1. Bands before (left) and after (right) IPP–FFT processing. Ridges were recovered and became discernable again in the right band. The band is from specimen Hgll-8-9(the fifth band).

The importance of the FFT could be easily understood in the following analysis. In most cases, a major problem in the interpretation is how to deal with some seemingly wide ridges (Fig S2). Poor preservation results in abrades peaks of adjacent ridges, making them look like an integrated whole. However, variations in the climate and solar radiation would also produce extra-saturated ridges. In addition, seasonal changes in the ridge width are also significant (Fig S3c). Therefore, the numbers of ridges in those situations are different.

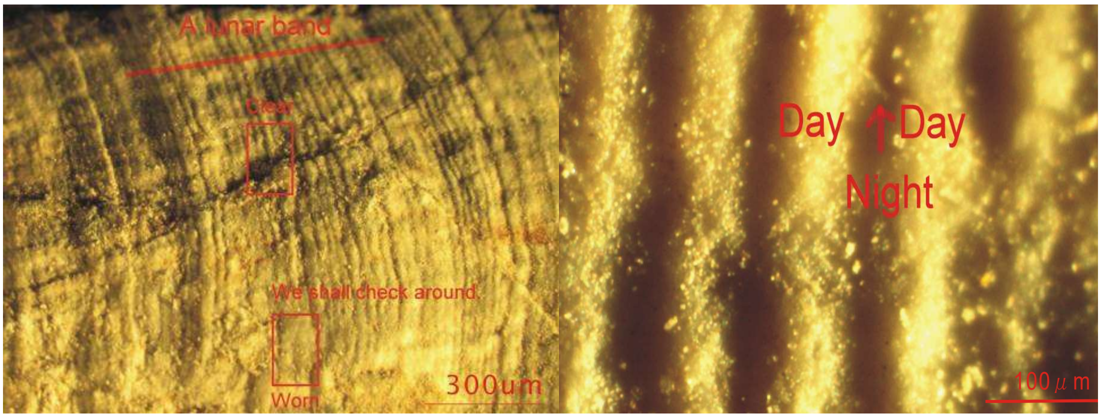

FigS2. This example of a lunar band under a stereoscopic microscope (80×) helps clarify our method of counting.

While scanning from the left border to the right border, the researcher comes across ridges that seem to be too wide, which is a location of wearing. By checking the surrounding area, we can find the clearest part of the same ridge and continue counting from there. Only the number obtained from the clearest areas can be recorded. Specimen name and depositary: DZ-30-16

To identify the actual number of ridges, biochemical measurements of recent corals in Guangzhou and a physical calculation were made to model clearly the process of daily calcium deposition. The calcification equation is

$$Ca^{2+} + 2HCO_3^{-} \xleftrightarrow{2photon} CaCO_3 + H_2CO_3 \quad \text{.} \qquad (S1)$$

To determine the impact of the light intensity on the saturating rate, calcification rates were measured under different light intensities at Sun Yat-sen University, China. The effect of light intensity on the calcification rates of *Stylophorapistillata* micro-colonies were presented in Fig S3a. Data suggested that the relation between the light intensity and calcification rate could be written as

$$y=0.00667x^2+0.433x+60 \qquad \text{(S2)}$$

where y is the calcification rate and x is the light intensity. On the other hand, in the present study, the daily solar radiation variation was also measured at Sun Yat-sen University, and the results are presented in Fig S3b. By combining the results, a relation between the time and daily calcification was derived using Equation (S2), as plotted in Fig S3c. Note that this was not the final pattern on coral epitheca because the daily traverse growth rate also depends on the light level and temperature. According to the study of [1] on *Galaxeafascicularis*, the coral's traverse growth rate in light is almost twice that under dark conditions. Hence, the final shape should be plotted as shown in Fig S3d.

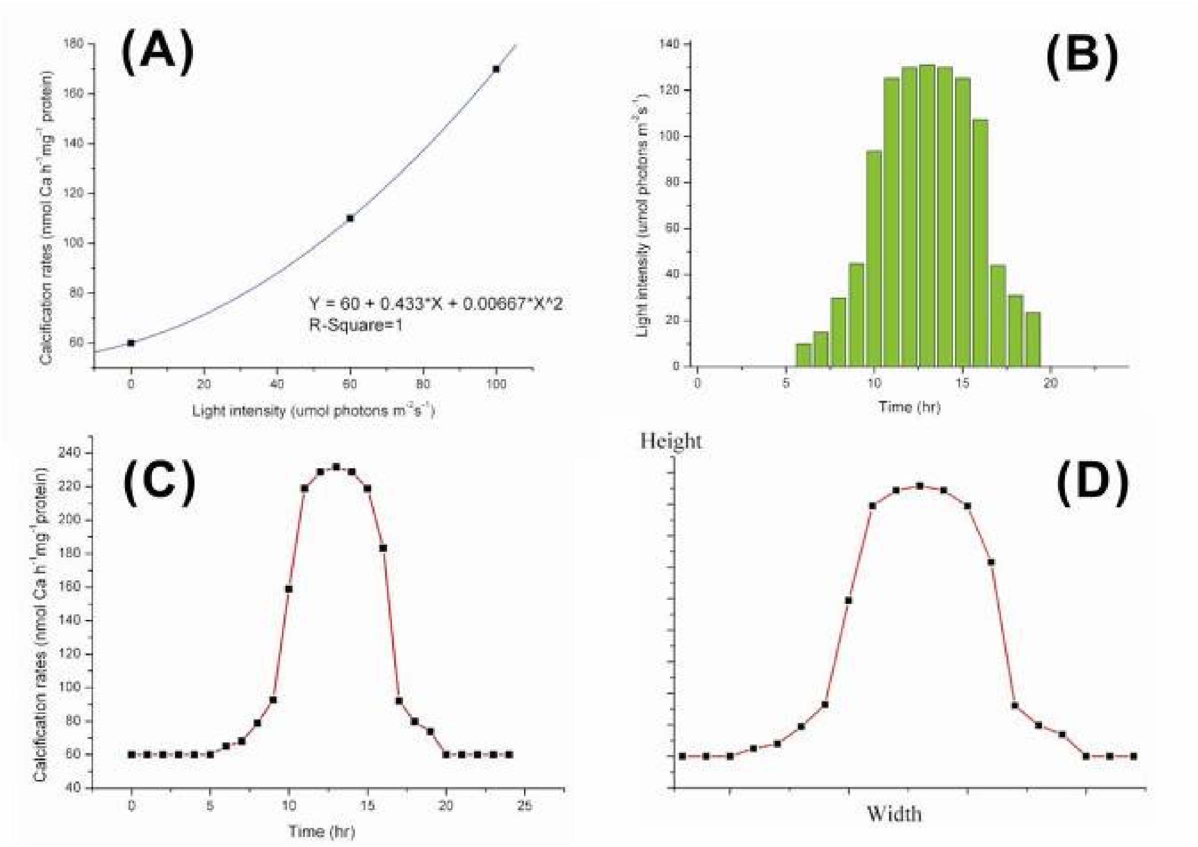

Fig·S3 (A) Effect of light intensity on the calcification rates of Stylophorapistillata micro-colonies. (B) Daily solar radiation variation measured at Sun Yat-sen University, Guangzhou, China. (C) Relation between time and daily calcification rate derived from $y=0.00667x^2+0.433x+60$. (D) Daily ridge model derived from analysis of coral light-enhanced calcification.

After applying the FFT, some wide ridges transform into two narrow ridges with the above theoretical shape.

(3) It is possible, even for very fine fossils, to be partly worn. However, ridge information could not be totally lost. Therefore, one should search for the best preserved ridge (usually the most closely grained) in each lunar band and have the precognition of this band or specimen. Then, according to the maximum counting rule, each potential ridge should be marked without overlapping. The number of ridges in each band was counted at approximately where the maximum count could be obtained. There should be no wide spaces left blank because ridges in the same band have

similar width and are consecutive. Therefore, while counting the ridges, we should search for residuals of another ridge. Simply counting in a direct line may lose information.

(4) IPP phase matching technique were used. All authorities agree that the skeletal density banding, whatever its cause, is an annual rhythm. However, a problem remains unsolved: the difficulty of determining where to start and end the counting of an annual pattern.

Here, by means of modern laboratory techniques and computer simulations, an ideal coral shape in a tropical area was calculated and found to show clear annual annulations without uncertainty. Therefore, by phase matching technique, we identified where corresponds to the start and the end.

Fig S4a and Fig S4b show the sea temperature variation in tropical oceans (the monthly tropical sea temperature variation obtained from the Japanese Oceanic Information Centre, Naha (26°12'N) and Ishigaki-jima (24°20'N)) and its relationship with coral skeletal growth. In addition, by fitting data from [2], a relationship between mean surface temperature (MST) and coral skeletal growth was determined (Fig S4c):

Growth rate=2.97×MST – 64.9 (S3)

In addition, the radius of the coral (if like a cylinder) also changes according to seasonal variations. We calculated the functional relation between the coral's growth density and MST from the ORIGIN fitting (Fig S4d):

Density=4.4 – 0.12×MST.(S4)

We simply suggested a secretion constant as 1, according to $V = \frac{m}{\rho} = \pi r^2 h$, where

V is the volume, m is the secreted mass, h is the width of lunar bands, and r is the coral radius. We then have

$$Coral\ Radius\ r = \sqrt{\frac{1/\rho}{h\pi}} = \sqrt{\frac{1/(4.4-0.12MST)}{\pi(2.97\text{MST-64.9})}}\ . \qquad \text{(S5)} \qquad \text{(Fig S4f)}$$

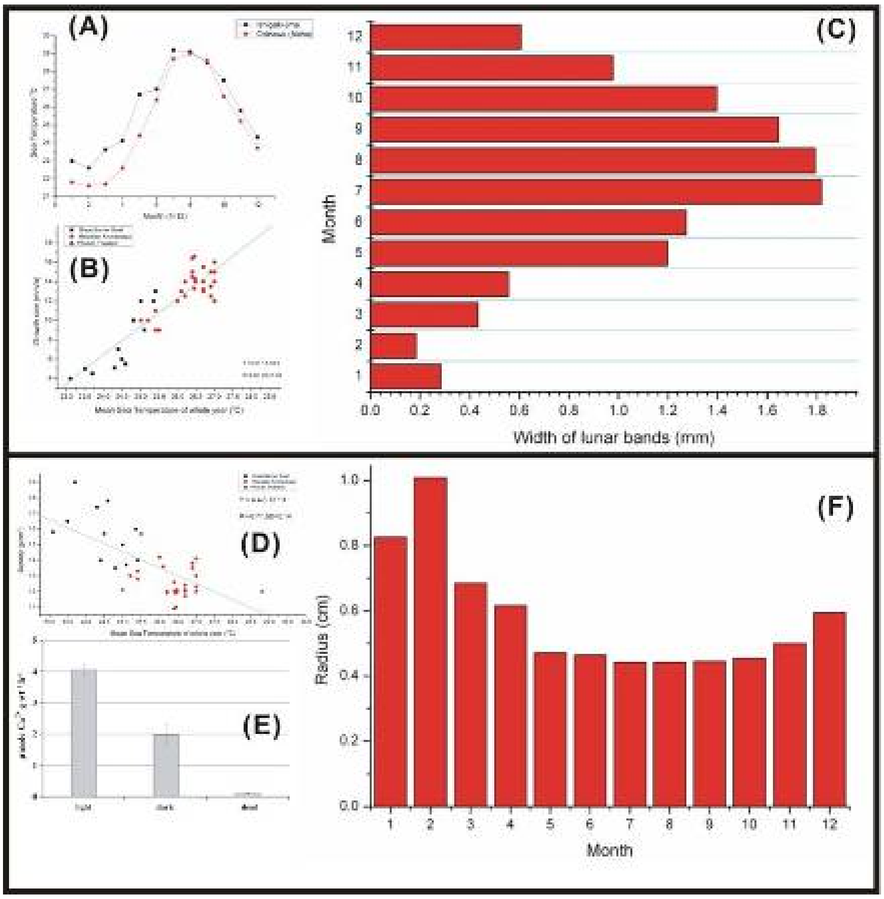

FigS4.Theoretical widths of lunar bands and coralradii based oncalculations. (A)Tropical sea temperature monthly variation from Japanese Oceanic Information Centre. Naha (26°12’N) and Ishigaki-jima (24°20’N). (B) Relationship between MST and coral skeletal growth. (C) Lunar band width model. (D)Recorded coral density under different MST from 29 reefs of the Great Barrier Reef, data averaged across colonies from 14 Hawaiian Archipelago reefs, data averaged across colonies from a reef at Phuket, Thailand [2]. (E) Calcification rates in the light, the dark and for dead colonies of Galaxeafascicularis from [1]. (F) Coral lunar radius model.

Our predicted annulation was somewhat protrudent. In fact, because of a lack of nutrition in winter, the secretion constant would be smaller than 1 in winter, resulting in less annulation than calculated and a better fit to the observations. However, the annulation was already sufficient for deciding where to start or finish counting in the

IPP phase matching method. This method compared fossils with a theoretical shape, disregarded noise, and was better than simple human estimation.

(5) The processing was checked again by another researcher and corrected as necessary.

(6) After the recording of data, we examined the results with the measurement of increment thickness; i.e., our counting result should be close to the approximation derived by the thickness measurement. We choose several clear ridges from the same band of the same specimen and measure their width. We then measure the band's width. Therefore, the approximation is the width of the band divided by the mean width of the ridges.

(7) Finally, the IPP graphic processing facility was used to check whether or not the result was in the confidence interval. IPP can provide frequency information of a specimen. Each peak reflects a height change on the epitheca.

References

1. Al-Horani FA, Tambutté É, Allemand D. Dark calcification and the daily rhythm of calcification in the scleractinian coral, Galaxeafascicularis. Coral Reefs, 2007, 26:531–538

2. Zhang J Y, Yu K F. Review on the Study of Coral Skeletal Growth (in Chinese).Geological Review, 2008, 54(3):362-372
