# Supplementary material for: Late paleozoic climate revealed by coral fossil patterns
Source: PLoS One. 2023 Aug 15;18(8):e0290127. doi: 10.1371/journal.pone.0290127 (PMC10426913; doi:10.1371/journal.pone.0290127)
Supplement: S2 File — (PDF) [file pone.0290127.s002.pdf]

S2 File. Specimen details

Specimen DZ-30-16(Late Carboniferous ~310Ma, Shiqiantan Formation, 16 bands)

(1) Ridges between two annulations (13 bands): 395(See enlargements below.); 30.4 ridges per band (Mean)

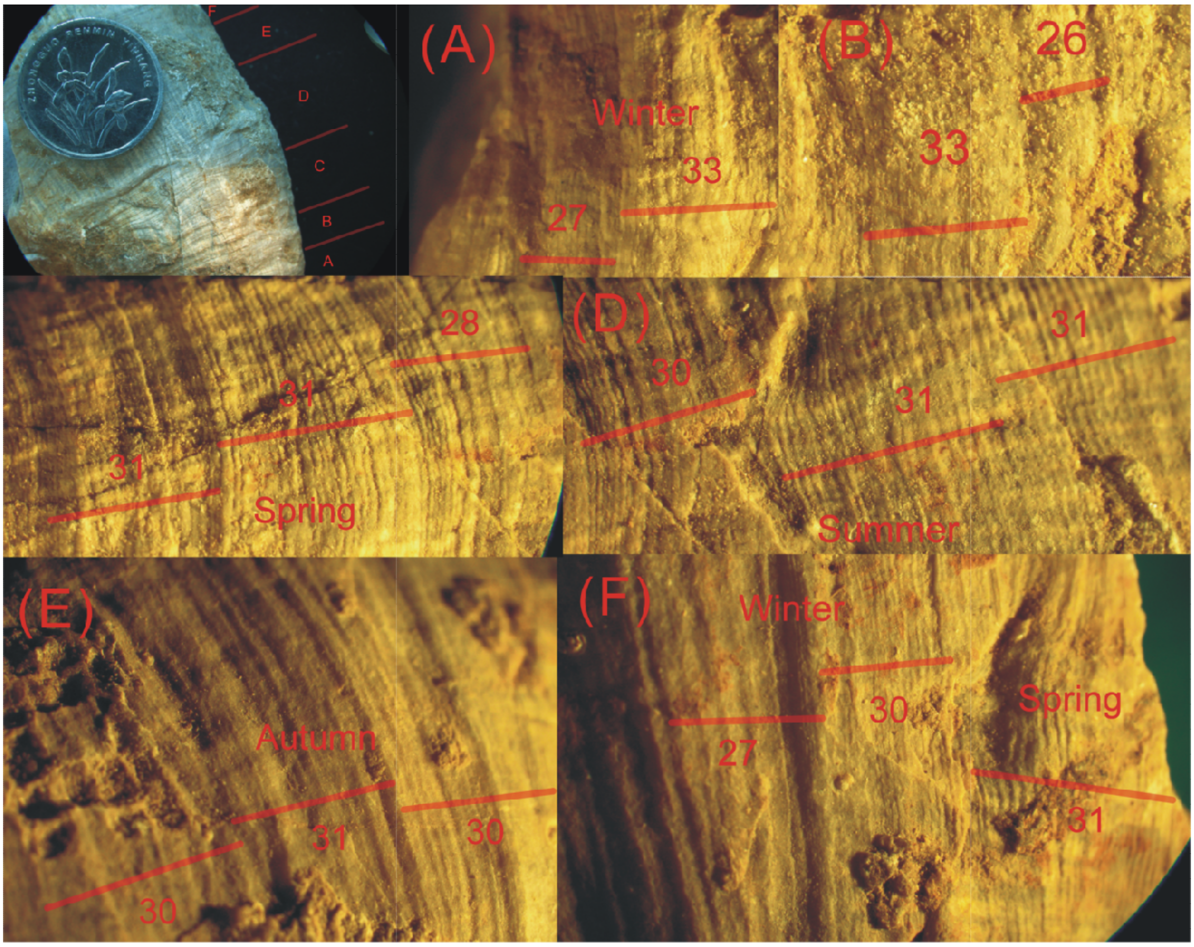

Fig S5. An overview of coral fossil DZ-30-16 and Enlargements of each part (containing 1~3 lunar bands) on coral epitheca.

Partial enlargement details are presented below:

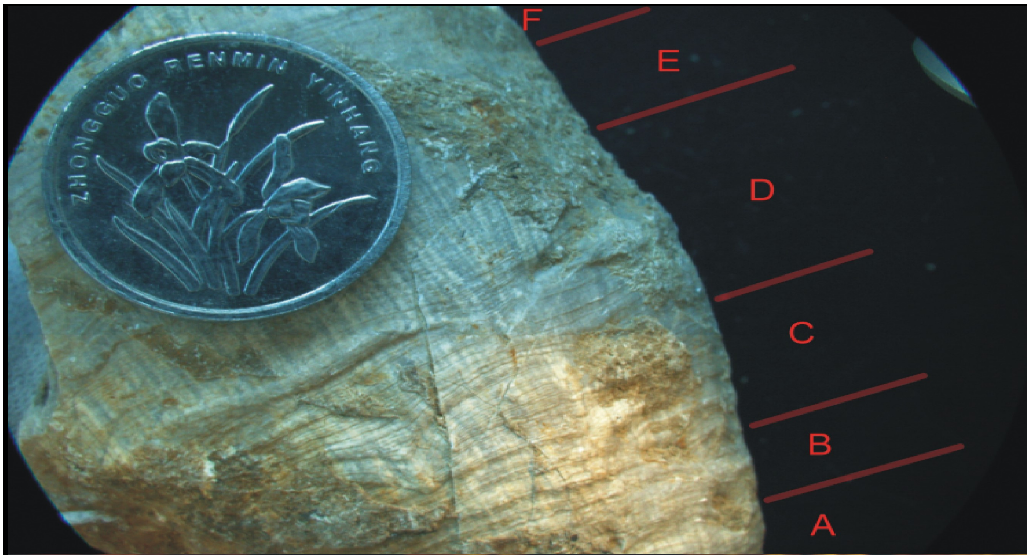

Fig S6. **Partial enlargement of coral fossil DZ-30-16.**

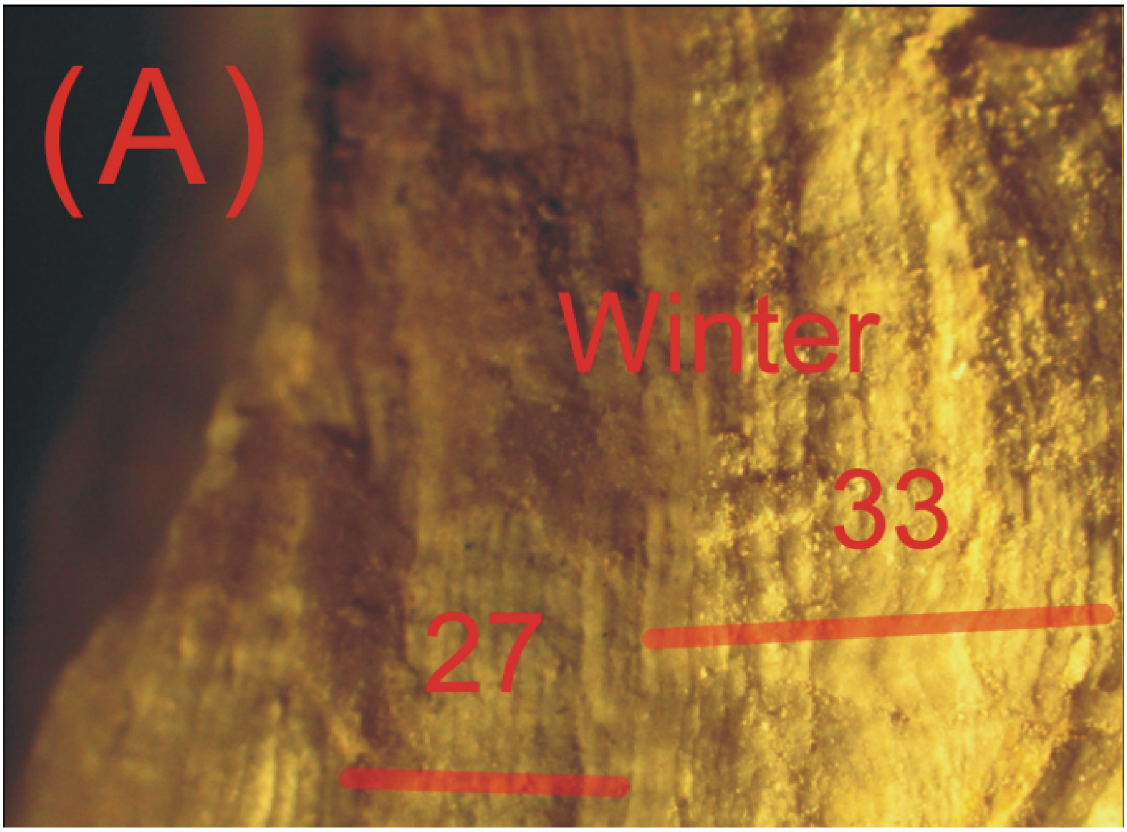

Fig S7. **Partial enlargement of coral fossil DZ-30-16.**

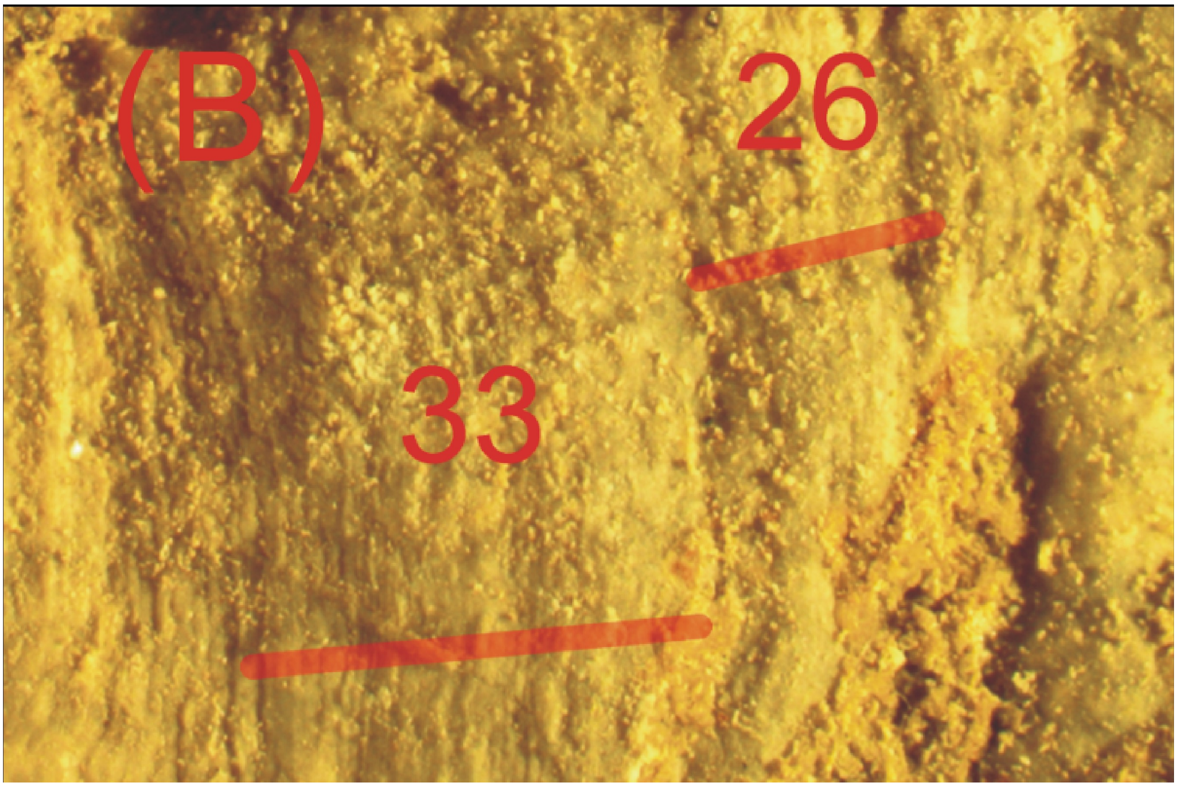

Fig S8. Partial enlargement of coral fossil DZ-30-16.

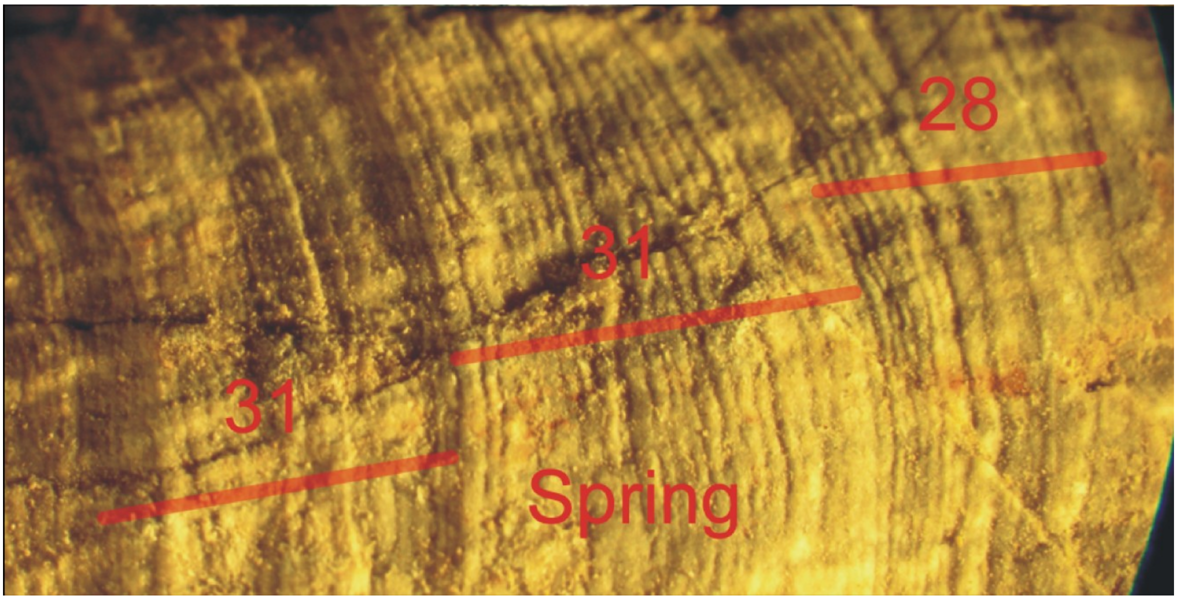

Fig S9. Partial enlargement of coral fossil DZ-30-16.

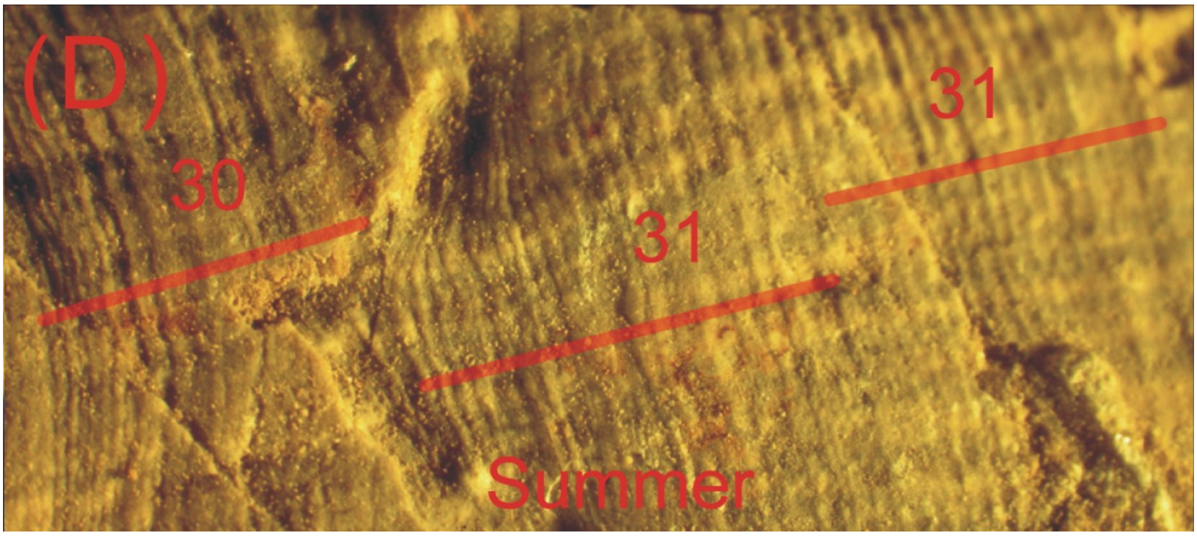

Fig S10. **Partial enlargement of coral fossil DZ-30-16.**

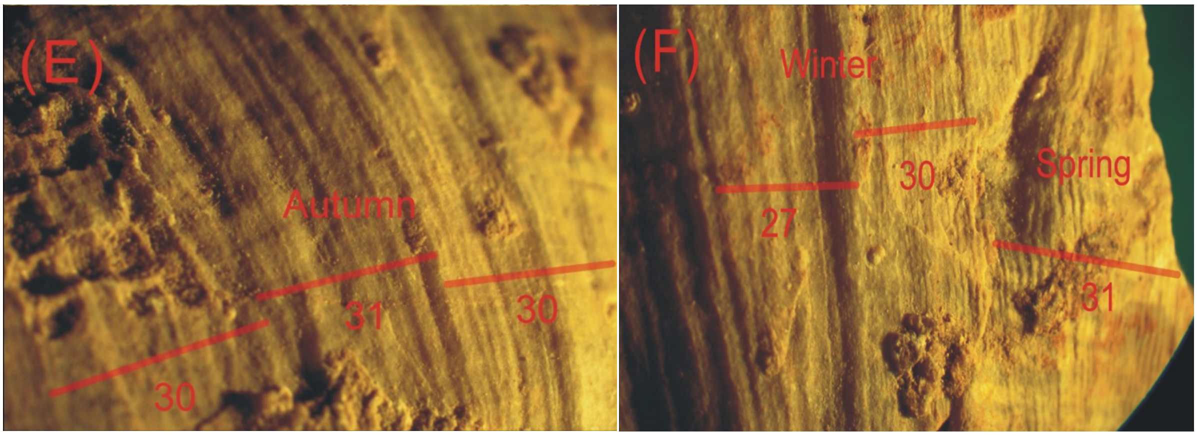

Fig S11. **Partial enlargement of coral fossil DZ-30-16.**

Specimen DZ-30-24(Late Carboniferous ~310Ma, Shiqiantan Formation, 18 bands)

(1)Ridges between two annulations: 394 (See enlargements below.); 30.3 ridges per band (Mean)

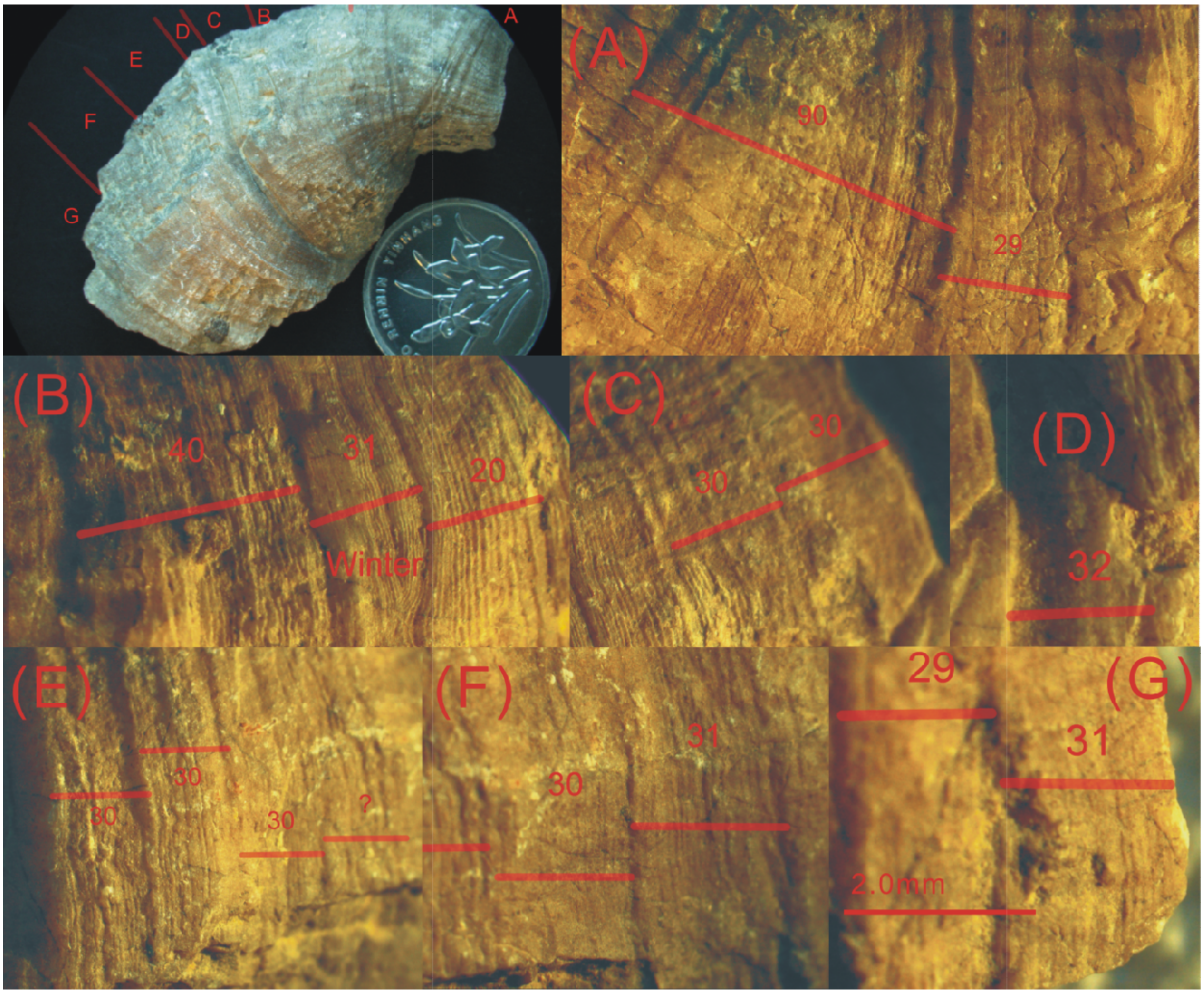

FigS12. An overview of coral fossil DZ-30-24 and enlargements of each part (containing 1~4 lunar bands) on coral epitheca.

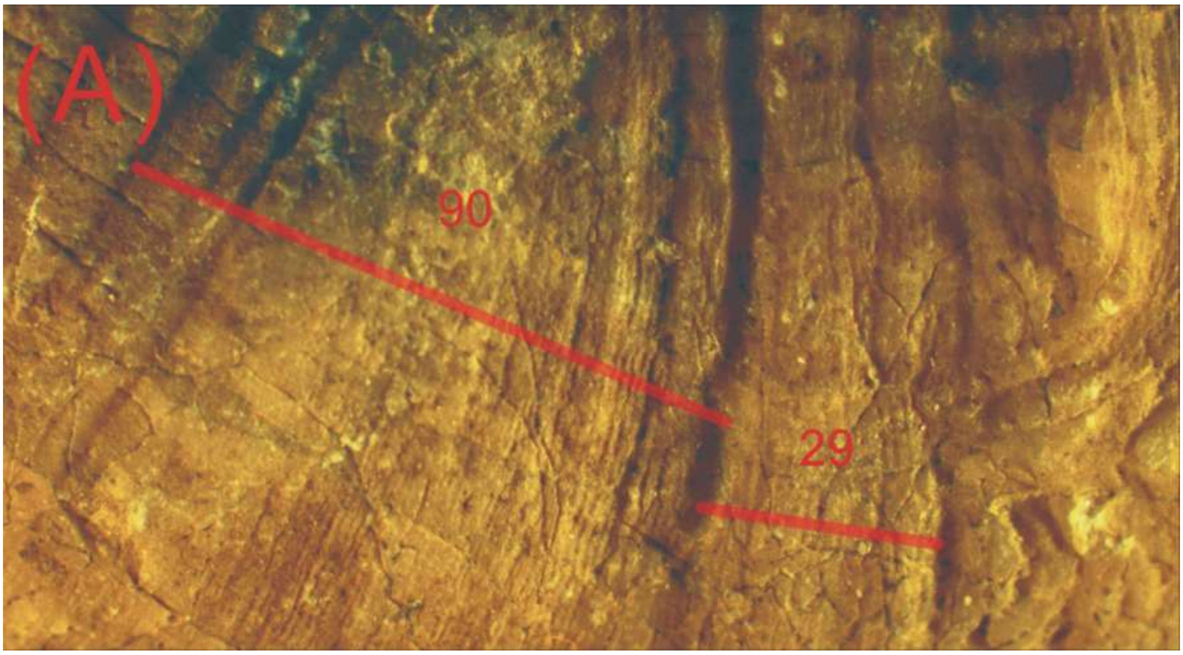

FigS13. Partial enlargement of coral fossil DZ-30-24.

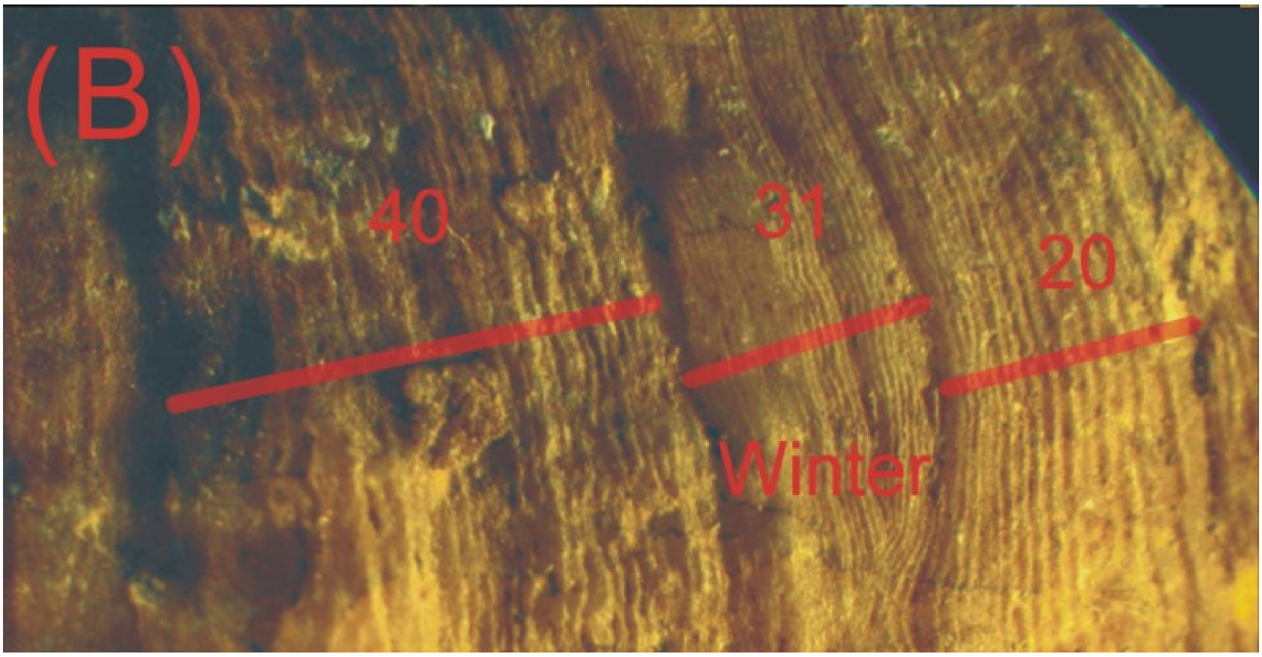

Fig S14. Partial enlargement of coral fossil DZ-30-24.

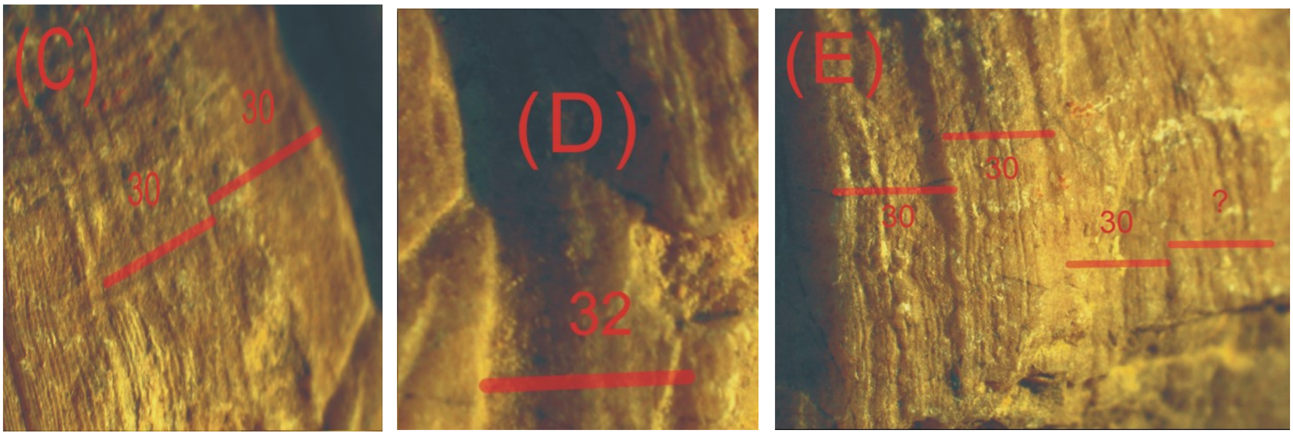

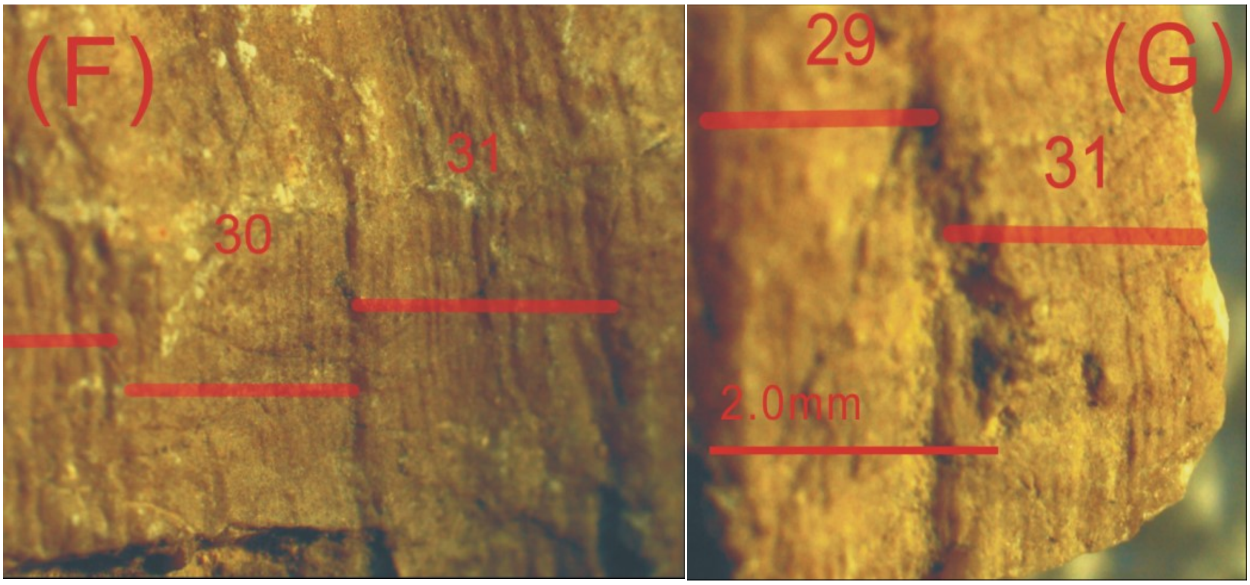

Fig S15. Partial enlargement of coral fossil DZ-30-24.

Specimen DZ-30-25 (Late Carboniferous ~310Ma, Shiqiantan Formation, 14 bands)

Ridges between two annulations: 394 (See enlargements below.); 30.3 ridges per band(Mean)

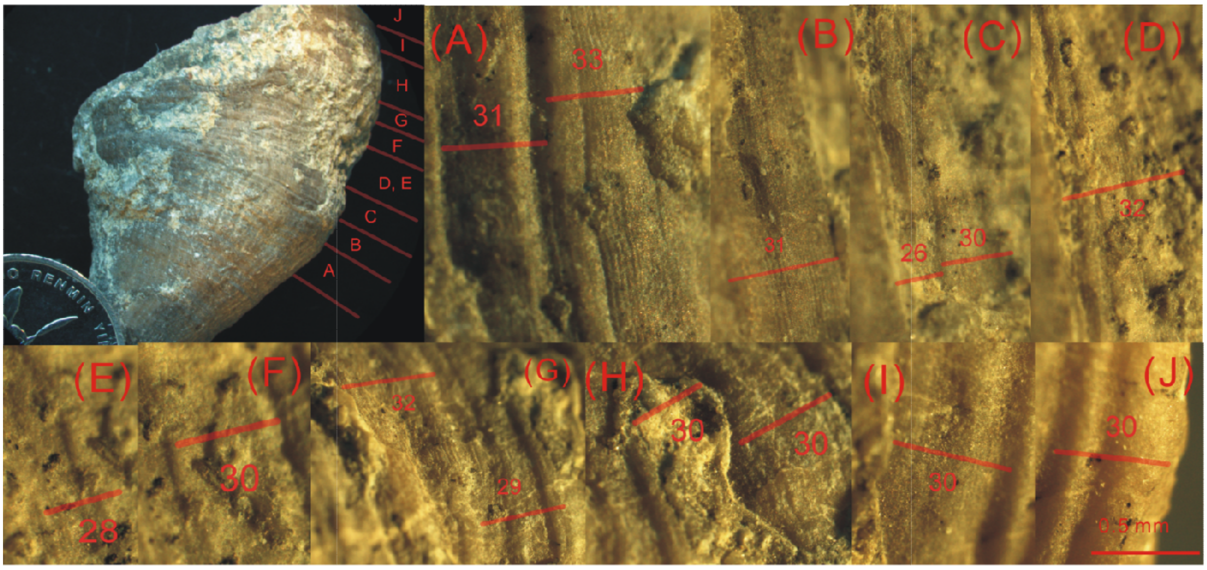

Fig S16. An overview of coral fossil DZ-30-25 and enlargements of each part. (containing 1~2 lunar bands) on coral epitheca.
