# Supplementary material for: Late paleozoic climate revealed by coral fossil patterns
Source: PLoS One. 2023 Aug 15;18(8):e0290127. doi: 10.1371/journal.pone.0290127 (PMC10426913; doi:10.1371/journal.pone.0290127)
Supplement: S3 File — (PDF) [file pone.0290127.s003.pdf]

S3 File. Confidence interval analysis

The Wilcoxon signed-rank test is a widely employed nonparametric statistical test used to compare differences between two correlated samples derived from the same individuals, but obtained under distinct conditions. It is commonly applied to analyze differences in data values collected at different time points. The fundamental principle of this test involves converting the difference between each paired sample into a signed rank, and subsequently assessing whether there exists a statistically significant difference between the two sample sets by comparing the sum of the signed ranks. One notable advantage of this method is its ability to accommodate small sample sizes and non-normally distributed data, as it does not necessitate stringent assumptions about the data distribution [1].

Fig S17 depicts the temporal variation in the width of the growth line of the DZ-30-16 sample. The graph illustrates distinct curves characterized by a continuous non-normal distribution, along with the presence of a noticeable seasonal trend.

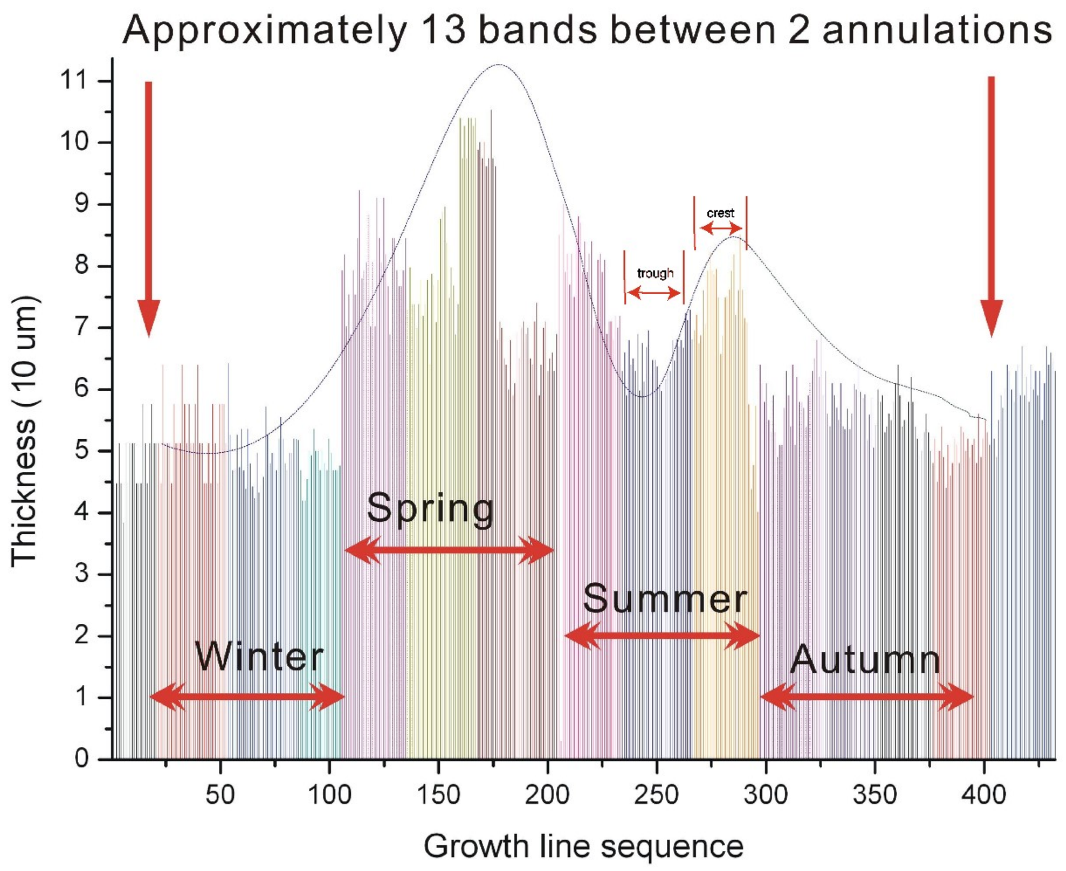

Fig S17. Curve of the Growth Line Width of the DZ-30-16 Sample over Time

To assess the significance of the differences between the trough and crest values of growth line widths in ancient coral fossils, we selected the trough and crest data labeled in Fig S17 for analysis. Specifically, we extracted the trough data corresponding to 20 growth lines (236-257) and denoted them asL_i (i=1,2,..,20), as well as the crest data corresponding to 20 growth lines (268-288) labeled asH_i (i=1,2,..,20). We then performed the Wilcoxon signed-rank test. Table S1 presents the corresponding numbers and growth line widths for the troughs and crests of the DZ-30-16 sample.

Table S1. Corresponding numbers and growth line widths of troughs and crests in DZ-30-16.

Trough growth	Trough growth line	Trest growth	Trest growth line

line number	number L_i (10μm)	line number	number H_i (10μm)
236	5.89492	268	6.93853
237	6.79662	270	6.86166
238	6.3709	271	7.5919
239	6.48916	272	7.05383
240	6.27038	273	7.89936
241	6.88236	274	8.20683
242	5.96292	275	7.89936
243	6.72863	276	7.86093
245	6.67837	277	7.93779
247	6.20239	278	7.4766
248	6.45664	279	6.5542
249	2.45664	280	6.86166
250	6.33838	281	7.4766
251	6.04865	282	7.51503
252	5.96292	283	7.5919
253	6.14917	284	7.89936
254	6.34134	285	8.16839
255	6.26447	286	7.20756
256	6.7848	287	7.5919
257	6.43594	288	8.51429

First, hypothesis testing was performed and the null and alternative hypotheses were constructed as follows:

Null hypothesis (H0): There is no significant difference between the trough and crest data of the DZ-30-16 sample.

Alternative hypothesis (H1): There is a significant difference between the trough and crest data of the DZ-30-16 sample.

The null hypothesis (H0) and the alternative hypothesis (H1) are two mutually exclusive hypotheses. The null hypothesis assumes that there is no significant difference, while the alternative hypothesis posits that there is a significant difference. Through data collection and hypothesis testing, we can determine which hypothesis is supported based on the statistical significance of the data [2]. In this particular case,

our aim is to investigate the presence of a significant difference between the trough and crest data of the DZ-30-16 sample.

Next, we computed the differences between the 20 sets of trough and crest data as $\Delta_i = L_i - H_i (i = 1,2,..,20)$. We then arranged these differences in ascending order to obtain a signed rank, ranging from 1 to 20. In cases where the difference was negative, a negative sign was assigned to the corresponding rank. Table S2 presents the results of calculating the differences of the DZ-30-16 samples and their corresponding signed ranks.

Table S2. Difference and signed rank of DZ-30-16 Sample.

**difference $\Delta_i = L_i - H_i$ (10μm)**	0.07	0.10	0.57	0.81	0.94	1.04	1.13	1.14	1.22	1.26
**signed rank**	1	2	3	4	5	6	7	8	9	10
**difference $\Delta_i = L_i - H_i$ (10μm)**	1.27	1.32	1.47	1.63	1.63	1.75	1.83	1.94	2.08	4.41
**signed rank**	11	12	13	14	15	16	17	18	19	20

Based on Table S2, the sum of signed ranks for positive values is a positive rank sum of 210, while the sum of signed ranks for negative values is a negative rank sum of 0. Based on these results, we can calculate the test statistic asmin{|positive rank sum|,|negative rank sum|} = 0.

Utilizing a significance level of 0.05 and a sample size of 20, by referring to the Wilcoxon signed rank test plot [3], we determine a critical value of 52. In order for the results to be considered significant, the calculated test statistic must be less than or equal to the critical value. As the test statistic of 0 is less than the critical value of 52, we reject the null hypothesis and accept the alternative hypothesis, indicating that there is a significant difference between the trough and crest data of the DZ-30-16

sample.

Therefore, at a 95% confidence level, we can conclude that the values of the troughs in the DZ-30-16 sample significantly differ from the values of thecrests. This indicates a high level of confidence (95%) in supporting the conclusion that there is a significant difference between the trough and crest data of the DZ-30-16 sample.

Similarly, the Wilcoxon signed-rank test was conducted on the DZ-30-24 sample to investigate whether there was a significant difference between the trough and crest data. Fig S18 below presents a plot depicting the width of the growth line for the DZ-30-24 sample over time, with the labeled trough and crest intervals. The labeled trough and crest data were selected for analysis in this study. Specifically, we selected the trough data corresponding to 20 growth lines (208-233) labeled as L_i (i=1,2,..,20), as well as the crest data labeled as H_i (i=1,2,..,20), and subjected them to the Wilcoxon signed-rank test. Table S3 displays the corresponding numbers for the troughs and crests of the DZ-30-24 sample, along with their respective growth line widths.

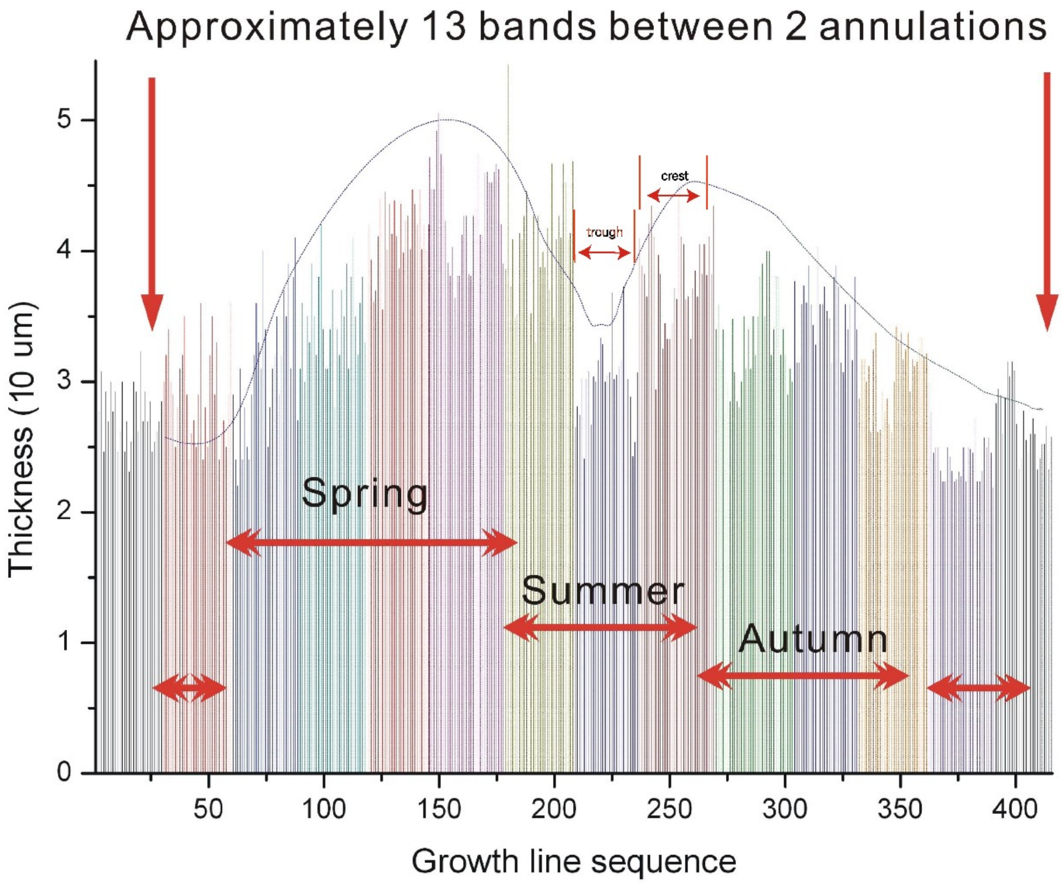

Fig S18. Curve of the growth line width of the DZ-30-24 sample over time

Table S3. Corresponding numbers and growth line widths of troughs and crests in DZ-30-24.

Trough growth line number	Trough growth line numberL_i (10μm)	Crest growth line number	Crest growth line numberH_i (10μm)
208	2.64449	236	4.08752
209	2.7972	237	3.88219
210	2.73152	238	3.82515
211	3.01135	241	4.3342
212	2.40316	242	4.09607
213	3.00525	243	2.93966
215	2.6616	244	3.95633
216	3.03805	245	3.81945
217	2.98956	247	3.4316
221	2.98956	250	3.4316
222	3.03805	251	3.70252
223	3.06229	253	4.49818
224	3.66972	254	4.09607
225	3.00525	255	4.03904

226	3.0466	260	3.84226
227	3.11219	261	3.64548
229	3.71108	262	4.04617
231	3.00525	263	3.81659
232	2.87407	264	4.064617
233	2.42348	266	4.10463

The results of calculating the differences of the DZ-30-24 samples and their corresponding signed ranks are shown in Table S4 below.

Table S4. Difference and Signed Rank of DZ-30-24 Sample.

**difference Δ_i=L_i-H_i (10μm)**	-0.07	0.33	0.43	0.44	0.44	0.53	0.66	0.78	0.80	0.81
**signed rank**	-1	2	3	4.5	4.5	6	7	8	9	10
**difference Δ_i=L_i-H_i (10μm)**	1.03	1.08	1.09	1.19	1.29	1.32	1.43	1.44	1.68	1.69
**signed rank**	11	12	13	14	15	16	17	18	19	20

Based on Table S4, the sum of signed ranks for positive values is a positive rank sum of 209, while the sum of signed ranks for negative values is a negative rank sum of 1. Based on these results, we can calculate the test statistic as min{|positive rank sum|,|negative rank sum|} = 1.

Using a significance level of 0.05 and a sample size of 20, by referring to the Wilcoxon signed rank test plot, we determine a critical value of 52. As the test statistic of 1 is less than the critical value of 52, we reject the null hypothesis and accept the alternative hypothesis, supporting the conclusion that there is a significant difference between the trough and crest data of the DZ-30-24 sample.

Therefore, at a 95% confidence level, we can conclude that the values of the troughs in the DZ-30-24 sample significantly differ from the values of the crests. This indicates a high level of confidence (95%) in supporting the conclusion that there is a significant difference between the trough and crest data of the DZ-30-24 sample.

References

1. Wackerly D, Mendenhall W, Scheaffer R L. Mathematical statistics with applications[M]. Cengage Learning, 2014.

2. Deng, J., Yang, W., Sutu, R., & Deng, Y. (2009). Probability Theory and Mathematical Statistics. Higher Education Press.

3. G Keller & B Warwick (1997) Statistics for Management and Economics (4th Edn), Journal of the Operational Research Society, 48:9, 963, DOI: 10.1057/palgrave.jors.2600936.
