# Supplementary material for: Late paleozoic climate revealed by coral fossil patterns
Source: PLoS One. 2023 Aug 15;18(8):e0290127. doi: 10.1371/journal.pone.0290127 (PMC10426913; doi:10.1371/journal.pone.0290127)
Supplement: S4 File — (PDF) [file pone.0290127.s004.pdf]

S4 File. Effects of obliquity of the ecliptic and precipitation on sunshine duration during the Late Carboniferous

According to the bimodal phenomenon of the growth line width of the coral fossil samples, we can infer that the late Carboniferous period of the sample has the characteristics of four seasons, indicating that the sample is located in a temperate zone. In addition, to form a temperate zone, the obliquity of the ecliptic must be less than 45°. According to Williams' high obliquity, low-latitude ice, strong seasonality (HOLIST) hypothesis [1], the Ediacaran period was during 635-590 Ma, and the obliquity of the ecliptic was about 54°.

Considering that the Late Carboniferous period of the sample is around 310Ma, just between the present and the Ediacaran period, we can perform a simple median calculation. That is, the obliquity of the ecliptic at that time may be between 38°21' and 39°30', which is less than 45 degrees. Therefore, the existence of temperate zones can be supported.

The obliquity of the ecliptic, solar zenith angle and local latitude are the main factors affecting the sunshine hours. In order to verify the accuracy of the estimated the obliquity of the ecliptic, we established the following model to calculate the sunshine hours [2-3].

$$\delta = \varepsilon - d*(\varepsilon*4/D) \quad (1)$$

$$N = T*(\arccos(\tan(-\delta * 3.14/180) * \tan(\varphi * 3.14/180.0)) * 180/3.14)/180 \quad (2)$$

Where ε represents the obliquity of the ecliptic, δ represents the solar zenith angle, d represents the number of days between the current date and the summer solstice, and the middle of the year is used as the summer solstice. φ indicates the latitude of the region, D indicates the number of days in the year, which are 384 days in the Carboniferous period ; and T indicates the length of a day.

Formula (1) is used to calculate the solar zenith angle, formula (2) is used to calculate the sunshine hours, and the unit is expressed in hours. Hence, we get a model for calculating the sunshine hours.

According to the formula (2), the theoretical sunshine hours of the Shiqiantan area during the Carboniferous period can be obtained, as well as its variation according to time. Assuming that the earth's revolution period remains constant it means that the length of a day in the late Carboniferous period is 22.3 hours.

According to Williams' HOLIST [1], it is estimated that during the late Carboniferous the obliquity of the ecliptic is 38 degrees, which is larger than the current value of 23°26′. Generally speaking, the increase of the obliquity of the ecliptic makes the sunshine hours longer in summer and shorter in winter.

On the other hand, the actual sunshine duration in the late Carboniferous was also affected by many factors, among which precipitation is an important factor. Precipitation was relatively high during the Late Carboniferous due to the presence of large rainforests and a humid climate. This high precipitation leads to cloud formation, which reduces the actual amount of solar radiation. Therefore, when studying the actual sunshine duration in the late Carboniferous, it is necessary to consider the influence of rainfall on the sunshine duration. This can improve the model of sunshine hours by adding rainfall factors to more accurately predict the actual sunshine hours. Therefore, the more precise sunshine hours model can be represented as following:

$$N=T^* (\arccos(\tan(-\delta * 3.14 /180) * \tan(\varphi * 3.14 /180.0)) * 180 /3.14) /180-p \quad (3)$$

Where, p represents the rainfall factor.

As a hypothesis, we use the current rainfall data of Beijing in North China to simulate the rainfall data at that time, and multiply it by a certain multiple. Among them, the simulation of 1.35 times the rainfall in Beijing is the closest. Based on model (3), the following figure is obtained:

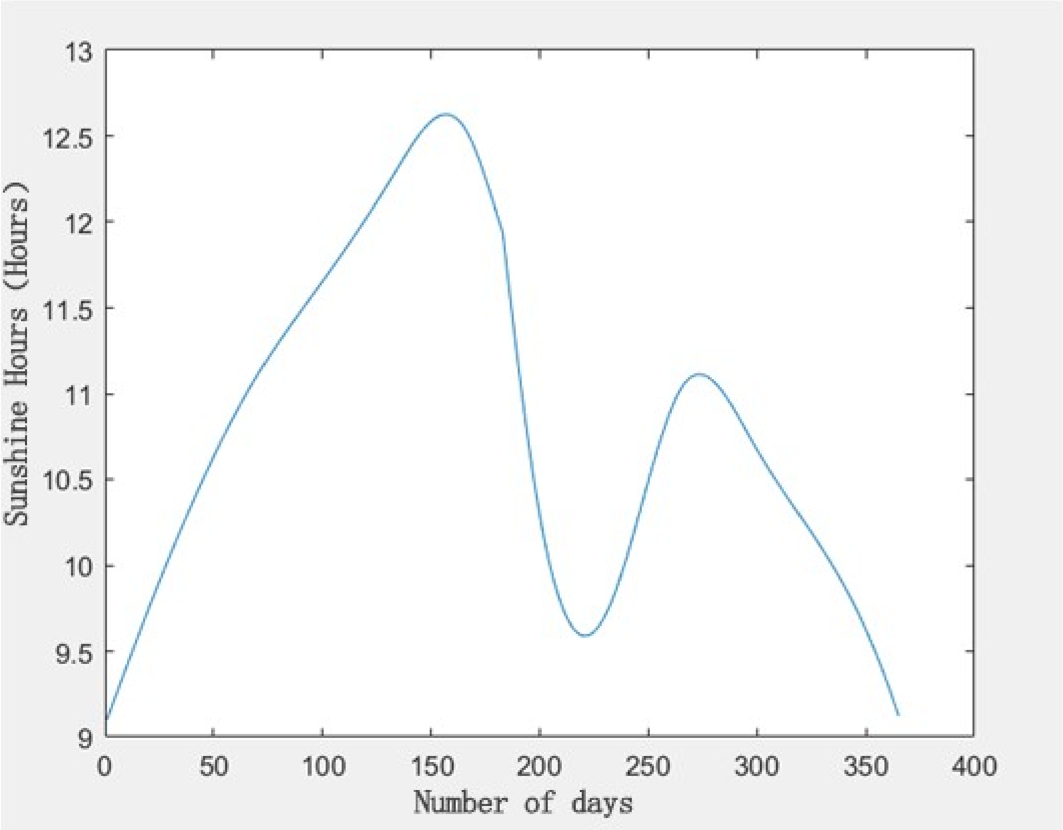

Fig S19. Bimodal shape derived from theoretical hypothesis.

Fig S19 is close to the graph obtained by actual statistics.

This matches the warm and humid climate of the Carboniferous period. The Carboniferous terrestrial organisms developed unprecedentedly, the climate was warm and humid, and there were swamps everywhere. There were plants over 40 meters long. Such lush vegetation must be inseparable from sufficient rainwater.

References

1. George E. Williams. Proterozoic (pre-Ediacaran) glaciation and the high obliquity, low-latitude ice, strong seasonality (HOLIST) hypothesis: Principles and tests [J].Earth-Science Reviews, Volume 87. Issues 3–4, Pages 61-93, ISSN 0012-8252.DOI:https://doi.org/10.1016/j.earscirev.2007.11.002.
2. Bourges B. Improvement in solar declination computation [J]. Solar Energy, 1985, 35(4): 367-369.
3. Woolf H M. On the computation of solar elevation angles and the determination of sunrise and sunset times

[M]. National Aeronautics and Space Administration, 1968.
